# Community hospitals – the place of local service provision in a modernising NHS: an integrative thematic literature review

**DOI:** 10.1186/1471-2458-6-309

**Published:** 2006-12-21

**Authors:** David Heaney, Corri Black, Catherine A O'Donnell, Cameron Stark, Edwin van Teijlingen

**Affiliations:** 1 Centre for Rural Health, University of Aberdeen, The Green House, Beechwood Business Park, Inverness IV2 2BL, UK; 2 Department of Public Health, University of Aberdeen, Aberdeen AB25 2ZD, UK; 3 General Practice & Primary Care, Division of Community-based Sciences, University of Glasgow, 1 Horselethill Road, Glasgow G12 9LX, UK; 4 NHS Highland and Department of Public Health, University of Aberdeen, Aberdeen AB25 2ZD, UK

## Abstract

**Background:**

Recent developments within the United Kingdom's (UK) health care system have re-awakened interest in community hospitals (CHs) and their role in the provision of health care. This integrative literature review sought to identify and assess the current evidence base for CHs.

**Methods:**

A range of electronic reference databases were searched from January 1984 to either December 2004 or February 2005: Medline, Embase, Web of Knowledge, BNI, CINAHL, HMIC, ASSIA, PsychInfo, SIGLE, Dissertation Abstracts, Cochrane Library, Kings Fund website, using both keywords and text words. Thematic analysis identified recurrent themes across the literature; narrative analyses were written for each theme, identifying unifying concepts and discrepant issues.

**Results:**

The search strategy identified over 16,000 international references. We included papers of any study design focussing on hospitals in which care was led principally by general practitioners or nurses. Papers from developing countries were excluded. A review of titles revealed 641 potentially relevant references; abstract appraisal identified 161 references for review. During data extraction, a further 48 papers were excluded, leaving 113 papers in the final review. The most common methodological approaches were cross-sectional/descriptive studies, commentaries and expert opinion. There were few experimental studies, systematic reviews, economic studies or studies that reported on longer-term outcomes. The key themes identified were origin and location of CHs; their place in the continuum of care; services provided; effectiveness, efficiency and equity of CHs; and views of patients and staff.

In general, there was a lack of robust evidence for the role of CHs, which is partly due to the ad hoc nature of their development and lack of clear strategic vision for their future. Evidence for the effectiveness and efficiency of the services provided was limited. Most people admitted to CHs appeared to be older, suggesting that admittance to CHs was age-related rather than condition-related.

**Conclusion:**

Overall the literature surveyed was long on opinion and short of robust studies on CHs. While lack of evidence on CHs does not imply lack of effect, there is an urgent need to develop a research agenda that addresses the key issues of health care delivery in the CH setting.

## Background

Recent policy developments within the National Health Service (NHS) in the United Kingdom (UK) have emphasised the need to bring services closer to home, with primary care increasingly acknowledged as the means of delivering population-based public health initiatives [1-4]. Such rhetoric needs to be matched with service re-design programmes that effectively and appropriately target health services nearer to the communities that they serve. This decentralised approach has been driven by a variety of factors including an aging population, difficulty in recruitment and retention of health care professionals and changes in working hours as a result of the EU (European Union) Working Time Directive, which stipulates the maximum working week for EU occupations including medical practitioners [5]. A range of service delivery models have been mooted, including nurse-led telephone services, on-line facilities, walk-in centres and the delivery of secondary care services (e.g. minor surgery, injury care and diagnostic facilities), in a primary or community-based setting. This has raised the profile of a neglected model of service delivery, the community hospital (CH), and led to decisions in the UK to reinvest in such a model of care [4,6,7]. However, the evidence to support their strategic role is unclear [8-11].

Explicit definitions of what a CH is, in terms of organisation, service delivery or public health function, were hard to find. Several currently exist, all derived from UK literature (Table 1). Common to all these definitions is the notion that care is led by family doctors, known as general practitioners (GPs) with their own GP beds, although consultant long-stay beds, primary care nurse-led and midwifery services may also feature, and that services are delivered locally to the patient. Beyond that, the services that may be offered are varied and can include not only inpatient facilities, but also outpatient, diagnostic, day care, primary care and outreach services for patients provided by multidisciplinary teams.

In light of renewed health policy interest in CHs, this lack of agreement on their role of and services raises a number of pertinent questions (Table 2). In order to address these, we conducted an integrative thematic review of the literature from 1984 to February 2005. This approach was chosen as an initial scoping of the literature highlighted the diversity of published evidence with many studies utilising qualitative approaches and few using comparative methodologies such as randomised controlled trials, rendering a Cochrane-style systematic review based on hierarchies of evidence inappropriate [12,13].

## Methods

We searched Medline, Embase, Web of Knowledge (including Science Citation Index & Social Science Citation Index), British Nursing Index, Cinahl, Health Management Information Consortium (HMIC), ASSIA, PsychInfo, SIGLE, Dissertation Abstracts, Cochrane Library Issue 3 and the King's Fund website [14] to identify published and grey literature. Databases (Table 3) were searched from 1984 to either December 2004 or February 2005, depending on availability. Table 3 lists the search terms, which included "community hospital", "cottage hospital" and "general practitioner hospital".

Utilising the definitions of CHs described in Table 1, we included studies or reports set in hospitals in which care was principally led by family practitioners (GPs) or nurses. Papers were also included in which visiting consultants provided secondary care or operating time in a CH. Hospitals with only specialist beds (e.g. geriatric long-stay) were not considered to fit the CH definition. However, social work establishments, such as a care home, were included if they also had GP beds. Diagnostic and treatment centres were not considered to be within the scope of this review, as they related more to the delivery of a secondary care function in a primary care setting. All study designs were included, as well policy documents and non-research based reports such as letters and editorials. Papers from developing countries were excluded, as were non-English language papers.

Two reviewers assessed each of the titles for eligibility. Disagreements were resolved by discussion. A third reviewer was consulted if consensus was not reached. Four reviewers then appraised the abstracts and keywords of potentially eligible papers for inclusion, scoring the reference according to suitability (0 – exclude, 1 – uncertain, 2 – include). Papers scoring 7 or 8, i.e. graded for inclusion by at least three reviewers and graded as uncertain by the fourth reviewer, were included in the final group of eligible papers.

Two reviewers abstracted data from eligible studies using a specifically designed data extraction tool previously piloted on 10 papers. Data extraction was in two stages: (1) details of the paper, setting, methods, aims and conclusions were recorded; and (2) each paper was assessed for content in relation to pre-defined themes based on the research brief and a preliminary review of the literature. These research themes reflected the questions posed in Table 2 and included: origin, number and geographical location; current role and place in health care provision; range of services offered and their effectiveness; interface between primary and secondary care; views of staff, patients and the wider community.

The development of the thematic review was guided by the methodological literature, which indicated that thematic analysis should involve the identification of prominent or recurrent themes, summarised under each thematic heading [12,13]. The content of these headings was used to identify and define overarching thematic categories, leading to a greater understanding of the topic. Thus, members of the research team read the relevant literature identified under each thematic focus, identifying both unifying concepts within each theme and discrepant issues. These were used to construct a narrative analysis for each theme. These were then reviewed across each theme, again to identify possible unifying concepts.

## Results

**Figure 1:**
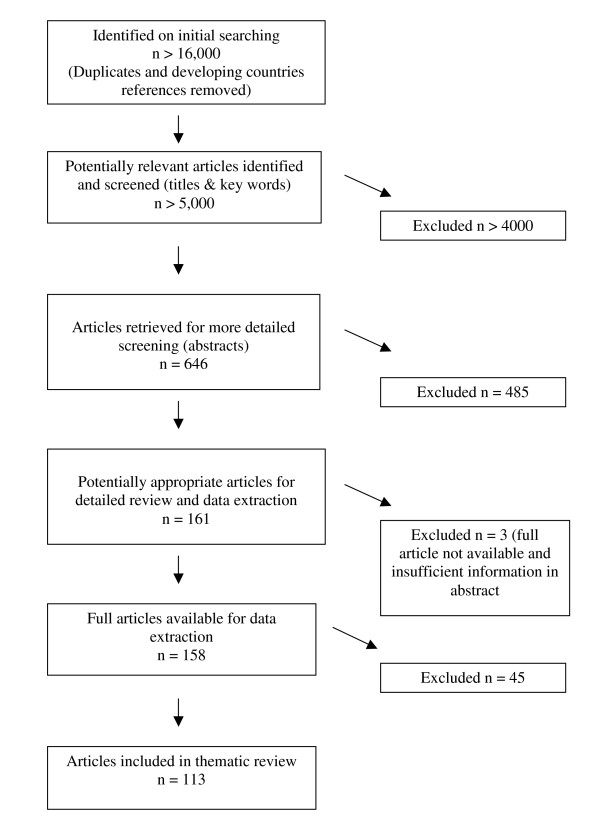
Selection process of eligible studies.

We identified 161 potentially useful citations. Full copies were obtained for 158 of these and 113 included in the thematic review (Figure 1). Of the 45 excluded, the main reason was that, on review, they were not primarily concerned with GP or nurse-led CHs. Most of the included papers originated from the UK, which may be a reflection of the UK definitions used to define CHs. While references from the USA were not specifically excluded, the criteria of being principally led by GPs or nurses led to the exclusion of most US literature. Most papers were descriptive or commentaries and there were very few studies employing: (a) an experimental design; (b) explicitly systematic reviews; or (c) economic studies (Table 4). Only one study reported on longer-term outcomes. Included studies are detailed in the additional file [see Additional file 1].

### Origin, number and geographical location

CHs have been a key part of health care provision both pre- and post-NHS, often evolving from cottage hospitals built in the latter part of the 19^th ^century. Planning of CHs was ad hoc, reflecting history rather than any rational planning [15-18].

Few reports detailed the current extent of CHs, either in terms of number or location. Two reported on the number of CHs in the UK, showing that numbers had increased from 455 in 1998 [18] to 471 in 2001 [19]. CHs appeared to be an integral part of health care provision in many rural areas [20], with Seamark reporting their mean distance from a district general hospital to be 21 miles for mainland Scotland and 14 miles for the rest of the UK [19]. GP beds were also a feature of health care provision in Finland, where most health districts had a "health station" with in-patient beds. While the overall use of these beds was 30% acute general medical care and 70% chronic or geriatric care, this proportion varied with distance from central specialist hospitals, with more acute care in the more remote areas [21].

As could be expected from Seamark's finding on distance [19], CHs were rare in urban areas, i.e. only four studies reported on three urban CHs (all in London) [22-25]. These provided acute medical care, observation, post-operative care, convalescence, rehabilitation and carer relief.

### CHs in health care provision

There was no single view of where CHs should sit in the continuum of care. For some, CHs were viewed as a step-down facility easing pressure on acute care services [26] and facilitating earlier transfer from acute hospitals [27]. Conversely, CHs could provide care more intensively than at home, but without moving patients to a higher intensity care setting, such as a district general hospital [26,28,29]. In this way, CHs functioned as an extension of primary care, contributing to acute, terminal and elderly care, as well respite care and rehabilitation services.

Several papers explored the role of CHs as local alternatives to secondary care, although these were mainly audits or cross-sectional surveys. An audit of surgery in CHs showed that surgeons operate in a CH setting with low rates of complications, but emphasised the need for liaison and co-operation between the CH and district general hospital [30]. CHs were also found to have a role in palliative care [31-33], with cancer-related deaths in other settings (district general hospital, hospice, nursing home or home) reduced where GPs had access to CH beds [32]. CHs were also used to provide cancer-related day surgery, including removal of skin cancers and breast excision biopsies, although it was unclear if this work was carried out by GPs or by hospital specialists working in the CH setting [33].

### Range of services offered and their effectiveness

A questionnaire to all CHs in the UK [19] identified a diverse range of services on offer (Table 5). Most reported studies focussed on obstetric care; geriatric care; accident & emergency (A&E) and unscheduled care (including the use of telemedicine); intermediate care; palliative care; and surgery.

#### Maternity care

We identified evaluations of one GP/midwife-led unit from the 1980s [34,35]. In terms of perinatal mortality, the unit compared well with the local maternity unit after adjustment for case-mix, but differences in standards of care in terms of monitoring and interventions were evident. A questionnaire of women cared for in a community maternity unit suggested that they were more satisfied with their care than women attending a district general hospital; however this study did not adjust for case mix nor did it present clinical outcome data [36].

#### Cardiac care

There was limited evidence from New Zealand (NZ) to support the ability of CHs to deliver acute cardiac care with the support of specialists [37]. Here, mortality following acute myocardial infarction was comparable to that of a large central hospital but, again, there was no adjustment for case mix. A German study of chronic cardiac care found that heart failure management according to current guidelines, use of beta-blockers and ACE inhibitors, and invasive cardiac examination was performed significantly less in the rural CH than in the metropolitan heart center [38].

#### Palliative care

An audit of services in one region of Scotland against palliative care standards developed nationally showed substantial variations between CHs and shortfalls against many of the standards [39].

#### Care of older people

This was often identified as a major function of CHs [18,26]. However, again, there was a marked lack of evidence of effectiveness. Two papers compared care of older people in CHs to that in larger district general hospitals [40,41]. Discharge rates, quality of life and mortality at 6 months were similar in both settings. However, an audit of nutritional services highlighted marked differences in care delivery in terms of care plans and observed practices between CHs and district general hospitals, with deficiencies noted in the CHs [42].

#### Use of telemedicine links

Telemedicine services were provided in some settings, mainly for Accident and Emergency (A&E) and minor injuries, linking GPs and minor injuries unit nurses to A&E specialists [43-47]. Again, there were no comparison groups to evaluate the effectiveness of the care provided.

### Efficiency of community hospitals

Efficiency was presented in the literature in terms of bed use, length of stay and cost, but not in relation to final health outcomes, e.g. life years gained or quality adjusted life years.

#### Bed use

Several studies described occupied bed days in CHs [48-51]. All reported reductions in admissions to general hospitals where GPs had access to CH beds (Table 6).

Round and colleagues [52] reported that, after adjusting for deprivation, other forms of residential care, distance from a district general hospital and morbidity, access to CHs accounted for 3.9 additional admissions per 1,000 population per year. So, while CHs may reduce admissions to other healthcare facilities, overall admission rates may be higher where CHs are available. However, no work examined the appropriateness of the admissions.

#### Length of stay

No studies compared length of stay in CHs with other models of care. There was limited evidence that length of stay was reduced when GPs co-ordinated the admission compared with consultants [27,53]. However, differences in case mix or appropriateness of care were not addressed.

#### Economic evaluation

Coast et al [54] estimated the cost saving (at 1994 prices) of an admission to a GP hospital at $72 – $144 (£47 – £94) per admission, with the potential to divert 5.6 – 8.4% of admissions to general medicine and geriatric specialties. McKinlay [40] reported savings of $49 – $62 (£30 – £38) per patient per day compared to a district general hospital at 1991 prices. A Norwegian study also concluded that the costs of GP hospital care were lower than district general care [55]. These cost-minimisation studies assumed equivalence of outcome.

Henderson and Scott [56] constructed a model of the economic impact of different patterns of care post-acute stroke and found that it was likely that admission to the CH in preference to a district general hospital would reduce overall costs of care. They concluded that it was possible to produce as good clinical outcomes in a community setting as in a district general hospital, given appropriate organisation of care. For a population of 13,500, the reduction (at 2000 prices) was approximately $165,408 (£109,000) per year. Their analysis was subject to numerous assumptions, but did suggest savings to the NHS. Costs to relatives were not examined; societal savings may have accrued by reducing travel costs to families.

### Equity

There was wide variation in access to CHs, across both rural and urban areas, even for people registered with GP practices with access to CH beds [51].

Equity does not only relate to geographical access: it also depends on the extent to which people with similar problems are treated in similar ways. However, most of the people admitted to CH in the studies reviewed were older, suggesting that admittance to CHs and the nature of care received was age-related rather than condition-related.

### Views of patients and staff

Most papers were cross-sectional, one-off surveys, using questionnaires of unproven validity. Studies were localised in nature, with small sample sizes.

Patient satisfaction levels with the care provided by CHs were usually high [25,30,36,44,57,58]. Benefits included ease of access, knowing staff and being cared for in a friendly atmosphere. Staff satisfaction was also high [36,58-60]. Benefits, according to staff, included convenience for patients, continuity of care, gain in knowledge and professional satisfaction. One exception was at a community midwife unit, where GPs were concerned about complications arising during labour and the risk this could present [36].

### Staffing

There were no papers focussing on the existing workforce or skill base, range of roles or training requirements of staff working in CHs. Table 7 shows the range of professional groups working in a CH setting, drawn from the identified literature.

There was evidence that professional boundaries were flexible in the CH setting, with role diversification apparent, especially for nurses [61-67]. This diversification of roles often challenged existing professional boundaries and, at its most extreme, could lead to breakdowns in communication with overall patient care suffering [62]. Opportunities for mutual learning and addressing structural and organisational barriers, such as inflexible contractual terms and conditions, can overcome these perceived divides [68]. The need for additional training and support was also highlighted in some papers, particularly for those practising in rural setting [47,69]. In Australia, joint academic posts between rural CHs and central academic units had been developed in order to improve recruitment and retention [69]

### The societal impact of CHs

There was no literature on the societal impact of CHs.

## Discussion

An extensive search of the evidence base for CHs identified a literature that was long on opinion and short of robust studies. However, it is important to stress that lack of evidence on CHs does not necessarily equate to a lack of effect.

The search and inclusion criteria were based on definitions of CHs as a setting where care is provided principally by GPs or by nurses. These definitions all originated from UK literature and so may be most relevant to health care systems organised in a similar manner to the NHS, where family practitioners act as a gatekeepers to specialist services, e.g. European countries such as Denmark and the Netherlands [70]. This focus on care led by GPs or nurses also led to the exclusion of a body of literature from the USA. However, there was literature from a range of other settings including Scandinavia and NZ. Thus, the findings are applicable to settings out with the UK.

There was no consensus as to where CHs sit in the continuum of care. They appeared to function both as a location to which patients requiring less intensive treatment could be discharged from acute care, prior to returning to the community; or as a site whereby more intensive care or respite care could be provided without resorting to admission to a more specialised hospital. Thus, CHs appear capable of functioning as a truly intermediate care site, providing care packages that are too complex for a patient to remain at home, but can also act as a location for patients who no longer require specialised care in the acute sector. This lack of consensus as to their location within the NHS may account for the lack of strategic direction and planning for CHs and also for the lack of detail regarding the organisational attributes and structure of existing CHs.

This also explains the range of services offered by CHs and the difficulty of judging the effectiveness of those services. Few studies adequately addressed the issue of effectiveness. The studies that did exist reported limited evidence of differences with specialised hospital care and outcomes for selected patient groups. However, comparisons were often limited and adjustments for case mix were not clear. Some studies appeared too small to identify any difference in outcomes. Differences in reasons for referral or admission to a district or specialised hospital rather than a CH, were generally not considered in studies. These weaknesses in the evidence suggest that it is not possible for opponents of CHs to mount a sustained argument that care is of a poorer quality in CHs than for the same groups treated in a district general or specialised hospital. Equally, there was little evidence that demonstrates equivalence of care in the two settings. However, this absence of evidence should not be taken as evidence of a lack of effectiveness. Like the ad hoc development and lack of planning of CHs, evaluation has been equally ad hoc. Lack of evidence of effectiveness reflects the lack of planned and systematic evaluation as well as uncertainty about the aims and role of the CHs.

Evidence of cost-effectiveness was even more sparse and based on the assumption that outcomes in CHs were equivalent to those in other settings. Of the two most detailed studies, one concluded that there was inadequate information to make a clear judgment, while the other argued that CH involvement would be cost-saving. Studies usually examined average bed day costs, and did not take into account fixed costs in district general hospitals that would not be offset by increases in CH activity.

Patients and staff appeared generally satisfied with care delivered in CHs, citing ease of access, continuity of care and knowledge of the staff as import factors. However, there was no discussion of the wider role that CHs may play in the society in which they are located. This is surprising, given their rural location, and the importance that health care practitioners may play in sustaining rural communities [71]. There was also almost no discussion of the future role that CHs may play in the provision of health care, for example as a location for unscheduled and out-of-hours care or as centres of telemedicine.

The challenges to research on CHs are substantial. Numbers are often low; randomisation is difficult or impossible; variation between hospitals is large; and there may be lack of agreement on appropriate outcomes measures. The nature of care provided in CHs makes measurement of outcome difficult, as measures should be generic, holistic and take a societal perspective. There are, however, numerous possible research routes to answer questions on clinical and cost-effectiveness, community impact and sustainability, combining qualitative and quantitative methods. We have thus identified a number of issues about the current variation in the structure and provision of CHs in different contexts, and their place in the future delivery of services (Table 8).

## Conclusion

The development of closer integration between health and social care, particularly in the UK, suggest that the strengths of CHs, linking primary and secondary care and providing a location for the delivery of complex packages of health and social care and public health, could be utilised further. This review indicates that research evidence on CHs could inform a clear national policy on their role, and benefit both the sector in its attempts to continue to adapt and health service planning in the NHS at large.

## Competing interests

The author(s) declare that they have no competing interests.

## Authors' contributions

All the authors designed the search strategies, contributed to the identification of eligible studies and subsequent data extraction and wrote the narrative analyses of individual themes. DH, CB and COD wrote the substantive drafts of the paper, with input from CS and EvT. All authors approved the final version of the paper. DH is the guarantor for the study.

## Funding statement

This study was funded by the Scottish Executive Health Department, Edinburgh, Scotland

## Disclaimer

The opinions expressed are those of the authors and not necessarily the funders or their employers.

## Ethical approval

Ethical approval was not required for this study.

## Pre-publication history

The pre-publication history for this paper can be accessed here:



## Supplementary Material

Additional file 1 Appendix 1. Summary of references included in the integrative review. This file details all 113 studies included in the review. Click here for file

## Figures and Tables

**Table 1:** Definitions of community hospitals.

"A general practitioner community hospital can be defined as a hospital where the admission, care and discharge of patients is under the direct control of a general practitioner who is paid for this service through a bed fund, or its equivalent." [8]
"A local hospital, unit or centre providing an appropriate range and format of accessible health care facilities and resources. These will include inpatient and may include outpatient, diagnostic, day care, primary care and outreach services for patients provided by multidisciplinary teams. Medical care is normally led by general practitioners in liaison with consultants, nursing and allied health professional colleagues, as necessary, Consultant long stay beds, primary care nurse-led and midwife services may also be incorporated" [9]
"Community hospitals are local hospitals, units or centres whose role is to provide accessible health are and associated services to meet the needs of a clinically defined and local population As an extension of primary care they enable GPs and primary health care teams to support people within their one communities. Community hospitals play a major role in rehabilitation and also offer palliative care, health promotion, diagnostic, emergency, acute and therapeutic services." [18]
"The general practitioner community hospital is one dominated by a primary care orientation in which patient selection, admission and management are all under the direct supervision of the general practitioner. These hospitals serve a confined geographical locality ...." [25]

**Table 2:** Questions considered by the review.

How common are community hospitals?
What is the range of services that provided by CHs?
What evidence exists about the effectiveness and efficiency of CHs?
What are they views of patients and staff?
What is the future potential of CHs?
What are the societal implications of CHs?
What is their impact on the wider healthcare system?

**Table 3:** Bibliographic databases searched and search terms employed.

**Database**	**Years searched**
Medline	1984 – February 2005
Embase	1984 – February 2005
British Nursing Index (BNI)	1984 – February 2005
Cumulative Index to Nursing & Allied Health (CINAHL)	1984 – February 2005
Health Management Information Consortium (HMIC)	1984 – February 2005
Applied Social Sciences Indexes & Abstracts (ASSIA)	1987 – November 2004
PsychINFO	1984 – November 2004
SIGLE	1984 – December 2004
Dissertation Abstracts	2003- November 2004
Cochrane Library	Issue 3, 2004
Web of Knowledge – including Science Citation Index and Social Science Citation Index	
King's Fund website	
	

**Search terms**	

Community hospital	
Cottage hospital	
General practitioner* hospital	
General practitioner* beds	
General practitioner* + hospital + bed	
Family practice + hospitals	
Hospitals – group practice	
Rural health + hospitals	
Family practice + hospital-physician relations	
Hospitals – community	
Hospitals – rural	

*GP abbreviation also used.

Full search strategies available from authors on request.

**Table 4:** Main methodological approach of included papers (n = 113).

**Methodological approach**	**Number of papers**
Systematic review	1
Randomised controlled trial	1
Controlled trial – not randomised	1
Other experimental design	1
Observational design (cohort, case-control, time series)	15
Cross-sectional survey, questionnaire	23
Case series, case study, audit	10
Descriptive	22
Qualitative research	3
Expert group opinion	3
Expert opinion (generally single author)	17
Economic study	3
Commentary/Non-systematic review	10
Unknown/Paper unavailable	3

**Table 5:** Services provided in UK community hospitals [18].

**Service**	**Number (%) of community hospitals (n = 471)**
Outpatient clinics	313 (66)
Minor injury units	330 (70)
Day hospitals	229 (49)
Physiotherapy	470 (100)
Occupational therapy	432 (92)
Speech therapy	361 (77)
Chiropody/podiatry	358 (76)
Inpatient & day care surgery	79 (18)
Maternity services	74 (16)^b^
Plain X-ray	296 (63)
Contrast X-ray	70 (15)
Ultrasound	151(32)

b. Of these, 20 units were solely midwife-led.

**Table 6:** Comparison occupied bed days in practices with and with and without access to general practitioner CH beds

	**Practices with GP Beds: Differences in Occupied Bed Days (Compared to Practices with no GP Bed Access)**		
	**Baker 1986**	**Hine 1996**	**Stark 2000**

Geriatric Bed Use	50% reduction	88.5% reduction	34.5% reduction
General Medicine		26.9% reduction	39.7% reduction
Surgery	-	1.6% reduction	18% reduction
Other	9.7% reduction	-	4.9% reduction

**Total**	**6.5% increase**	**7.6% increase**	**6.5% increase**

Note: Baker compared practices in Oxford City with no access to GP beds to other Oxfordshire Practices with no access to GP beds, to Oxfordshire Practices with access to GP beds. Analyses were divided into General Medicine and Geriatrics (one category); other specialties and all specialties in DGHs or GP hospitals. The results presented are comparisons of non-Oxford City Practices with Practices with GP bed access. (Source: Stark 2000)

**Table 7:** Professional groups working in a CH setting

**Professional group**	**Type of staff**
Doctors	GP (rural, urban) Specialist GPs (surgical, anaesthetic, obstetric palliative care, gynaecology etc.) Clinical assistants Visiting medical or surgical consultants Remote telelink support from A&E medical staff Community paediatricians Anaesthetists Emergency back up from multidisciplinary "flying squad"
Nursing	Extended role nurses Nurse led units Midwives Emergency nurse practitioners
Allied Healthcare professionals	Occupational therapists Physiotherapists Speech therapists Dietician
Other	Chiropodists Radiographers Social workers Ambulance personnel for case triage Other consultant specialities Acute care assistants

**Table 8:** Future issues for CH delivery and evaluation.

Is the variation in the composition of CHs acceptable?
Does the care provided in CHs map against local health care need?
Have CHs been constructed around the skills available in the local health economy, rather than the needs of patients?
Can delivery in CHs adapt or are they inflexible as structures in the local health economy?
Do CHs have a place in the resign of services?
Can they act as a buffer against the centralisation of care?
There are large parts of rural Scotland without CH provision. Is that acceptable?
Could these areas be used for comparative studies?
Are there other methods of bolstering the delivery of primary care in rural areas?
Do primary care beds need to be located in CHs?
Is there a role for urban CHs?
