# Supplementary material for: Community hospitals – the place of local service provision in a modernising NHS: an integrative thematic literature review
Source: BMC Public Health. 2006 Dec 21;6:309. doi: 10.1186/1471-2458-6-309 (PMC1769373; doi:10.1186/1471-2458-6-309)
Supplement: Additional file 1 — Appendix 1. Summary of references included in the integrative review. This file details all 113 studies included in the review. [file 1471-2458-6-309-S1.doc]

Appendix 1. Summary of references included in the integrative review.

| **Ref no** | **Author and Title** | **Year** | **Country** | **Main method (additional methods)** | **Data collection /source** | **Describe setting -  geographical, staffing, size etc** | **Main stated aims** | **Main Conclusions** |
| --- | --- | --- | --- | --- | --- | --- | --- | --- |
| [1,1] | Aaraas, I., Melbye, H., Eriksen, B. O., and Irtun, O. Is the general practitioner hospital a potential 'patient trap'? A panel study of emergency cases transferred to higher level hospitals | 1998 | Norway | Case series | Panel review  hospital case records | Finmark, the northernmost and most sparsely populated county in Norway. Has 16 community hospital with travelling time to district general hospital between 1 to 4 hours. Case histories from 73 patients (out of 395 consecutive patients admitted over 8 weeks) transferred as emergencies to higher level hospital were reviewed by a panel of 3 experts. | To assess whether a stay in a local GP hospital prior to emergency admission to a higher level hospital led to worse outcomes than direct admission and to detect cases where early treatment in the GP hospital might have had benefits. | There appeared to be few negative effects due to a transitory stay in the local GP hospital and these were moderate and acceptable comp+L6ared with more advanced wards. Negative health effects were balanced by the benefits of early access to life saving treatment in GP hospitals for critically ill patients. |
| [2] | Aaraas, I., Sorasdekkan, H., and Kristiansen, I. S. Are general practitioner hospitals cost-saving? Evidence from a rural area of Norway | 1997 | Norway | Economic | Cost minimisation | Finmark, the northernmost and most sparsely populated county in Norway. Has 16 community hospital Problems are long travel distances (up to 320 km from nearest hospital) and tough climate.  Full-year throughput of patients extrapolated+J5 from 8 week period with 415 consecutive admissions. | To compare the costs of GP hospitals with alternative forms of care (general hospital, nursing home, or home care) | GP hospitals are likely to provide health care at lower costs than alternative forms of care. |
| [3] | Anonymous. Return of cottage hospitals in search for more beds | 2000 | UK | expert group opinion ( brief press statement) | N/A | England | Hospital bed inquiry reported in 2000 need for additional inpatient beds in England | The authors suggest "resurrecting cottage hospitals" |
| [4] | Anthony, D. and Brooks, N. Clinical guidelines in community hospitals. | 2001 | England | Qualitative | Interviews | 24 F and G grade nurses, occupational therapists and physiotherapists in Leicestershire and Rutland healthcare NHS trust | To examine the use of clinical guidelines and attitudes towards them in community hospitals through in-depth interviews with purposive sample of staff | Most respondents were in favour of clinical guidelines and felt that more were necessary. Awareness and implementation of guidelines is hindered by barriers to education - IT support, resource rooms and use of link nurses etc. |
| [5] | Antrobus, M. Developments work in community hospitals. | 1996 | England | Descriptive | N/A | Andover. 102 bed hospital, containing a GP unit. 24 hour nursing, district nurse involvement | Description of development work, mainly around nursing roles, including district nurses | Change management difficult in any setting. Hierarchical hospital nursing staff arrangements may hinder change. District nurses can take a care management role, and beds can be used as nursing beds, with full admission and discharge rights. |
| [6] | Antrobus, M. Professional viewpoint. Community hospital nurses: raising the profile | 1996 | England | Expert opinion | N/A | Andover. No description of specific hospitals - lists 'examples of services provided in community hospitals'. | Description of educational activities in community hospitals, including integration with other training. | Community hospitals need increasingly complex skills. Provide good site for undergraduate and postgraduate education. |
| [7] | Archibald, G. Patients who have had a stroke: where should their needs be met? | 1998 | England | Descriptive | N/A | CH in West Yorkshire with 18 beds. | To determine the feasibility of the CH providing adequate care for patients who have had stroke. Also discusses factors involved in the care of stroke victims and the role of CHs in this care. | CHs may be an appropriate option for stroke aftercare due to: Focus as a provider of intermediate care with closer access and links to the community and to primary health teams. Multidisciplinary approach to rehabilitation Access to appropriate services and specialities. Also more cost effective |
| [8] | Armstrong, I. J. and Haston, W. S. Medical decision support for remote general practitioners using telemedicine | 1996 | Scotland | Cross-sectional survey | Questionnaire | Remote community hospital, Peterhead, 60km north of Aberdeen; Pop approx 20,000; Transport connections to Aberdeen poor: typical car journey 45-60min; public transport 75-90 min; Provides 24hr casualty service; GPs supply medical cover incl on-call doctor for casualty dept; Casualty dept treats approx 1300 patients/mths; radiology service provided during normal working hrs, outside of which non-acute trauma patients asked to return; Acute trauma patients attending at night transferred to Aberdeen by ambulance | Evaluation of telemedicine link between small community hospital and large trauma centre | Authors conclude that survey results indicated that both GPs and A&E consultants felt that teleconsultation had improved patient care. Discussion section highlights some problems with equipment limitations and some issues of quality; could be issues of disruption to departmental routine for A&E consultants; Consultations were GP led raising questions about responsibility and highlighting need to consider best practice in methods of medical telecommunication. |
| [9] | Ashworth, M.; Nafisa, M.A.; Corkery, M. Respite care in an intermediate care centre: the views of patients and carers | 1996 | England | Survey | Questionnaire | Community care centre in Lambeth (20 in-patient beds, 4 of which respite care beds). | To examine respite care for individuals who have physical dependencies through questionnaires. | 40 patients and 21 carers participated. Satisfaction with care ratings were high. Majority of patients felt the care had helped them to stay at home. Most carers felt the respite care had helped them physically and psychologically. Authors conclude that although the outcomes of this study were generally positive, these findings conflict with those from other studies. They believe this may be due to the fact the study focussed on patients with physical dependencies (as opposed to dementia) and also attribute positive findings to high use of the multidisciplinary team. |
| [10] | Baker, J., Goldacre, M., and Muir-Gray, J. A. Community hospitals in Oxfordshire: Their effect on the use of specialist inpatient services | 1986 | England | Observational | Hospital data | Oxfordshire Health District - population 503,000. 10 community hospitals (9-79 beds) with a total of 315 beds | To examine the association between GP access to community hospitals with use of DGHs and overall utilisation rates by looking at Hospital Access Analysis | Access to CH beds is associated with low rates of use for DGH beds. Authors concluded that CHs are used as a substitute for DGHs. Total utilisation rates are higher for populations with CH access. Authors present several possible explanations for this. |
| [11] | Barker, L. C. and McCarthy, S. T. Geriatric day hospitals: consultant and community units compared. | 1989 | England | Descriptive (Case series) | Hospital data | Oxfordshire. Comparison of a DGH geriatric day hospital with five GP run community hospital-based day hospitals in the catchment area of the same DGH. Staff not described in detail, but some OT and physiotherapy availability. | Description of activity at the two types of site, and comparison. | Similar age group, and similar dependency scores of service users. Higher number of beds / 1000 population > 75 years in GP Community Hospitals (GPCH). Higher proportion of new patients at DGH than GPCH. Longer median length of attendance at GPCH (332 days vs 93 days). More attendances at DGH (related to length of stay). Higher proportion of people discharged at six months at DGH (67% vs 35%). Very few people admitted to DGH from areas with GPCH. |
| [12] | Baxter, E., Bushell, A., and Pearson, V.Community hospitals. Delivering the goods | 1997 | England | Survey (Qualitative) | Questionnaire  Interviews | Three Community Maternity Units (CMU) in Honiton, Tiverton & Okehampton, Devon: total of 19 beds; Tiverton had back-up of two GP obstetricians & 3 GP anaesthetists, and facilities for surgical deliveries. Other two CMUs at Honiton & Okehampton largely midwife-led and where intrapartum care by GPs has declined. | Main purpose to compare perceptions of GPs, midwives & women regarding choices of maternity care and role of CMU. | Health professionals and women alike highly value CMUs;  Over half women in survey wanted to deliver at local CMU; reasons included conveniences, friendly atmosphere & flexible supporting staff more likely to be known to woman. Women felt more in control of birth experience, reported more satisfaction with postnatal care & support.  Health professionals views varied: GPs more concerned than midwives about risks involved with CMUs should complications arise, particularly when unit midwife-led. For this reason some GPs not happy to recommend CMU and some directly opposed, but no evidence that CMUs less safe; Relationships between professionals poor, service offered women suffered as a result; misunderstanding of roles & responsibilities, highlighting need for training and education; |
| [13] | Henderson, J. Benefits and costs of community and long-stay health services in the Borders | 1986 | Scotland | Economic |  | Option appraisal of health service re-provision in the Borders, including a GP unit. 25 bed hospital in Hawick. | Cottage hospital part of an option appraisal for service re-provision. | Health economic assessment of options for re-providing beds. |
| [14] | Berkeley, J. S. The role of the cottage hospital in the UK | 1983 | UK | Commentary / Non-systematic review | Literature / expert | Historical review of the role of the cottage hospital in the UK with data from Grampian presented | To outline the purpose of cottage hospitals | Role must relate to the population it serves |
| [15] | Brebner, E. M., et al Evaluation of an accident and emergency teleconsultation service for north-east Scotland | 2004 | Scotland | Descriptive (survey) (qualitative) | Questionnaire interviews | 14 community hospitals in north-east Scotland linked via telemedicine link with Accident and Emergency Department in Aberdeen. Community hospitals provide casualty service to local community. Each hospital was provided with at telemedicine link (videoconferencing facility that allowed transmission of radiographs) that provided medical advice from medical staff in A & E in Aberdeen, 24 hours /day, 7 days/week. Project included funding for three clinicians based in A & E Aberdeen (1 half-time consultant, two full time clinicians at SHO/registrar level).  Total of 1392 teleconsultations during 12 month period starting September 2001. | To evaluate an accident and emergency teleconsultation service provided to 14 community hospitals in north-east Scotland. | Sets out 10 recommendations based by the evaluation. Considers that telemedicine service has to be needs driven, sufficient workload required to maintain skills and confidence of staff, has to be a commitment from the main centre, telemedicine champions have to be identified, clear protocols, reliable equipment and communication structure, user-friendly equipment, training, staffing and training implications of treating patients locally need to be considered and the service has to be objectively evaluated. |
| [16] | Brockbank, J. What relevance do community hospital beds have for palliative care patients? | 2002 | UK | Expert opinion | N/A | No setting | To examine the role of community hospitals in providing palliative care for patients. | Good communication is essential for every form of health care especially palliative care. Community hospitals are relevant for patients requiring palliative care. |
| [17] | Brooks, N. Length of stay in community hospitals | 2001 | UK | Audit | Hospital patient data | Eleven community hospitals within one trust area. Total of 397 inpatient beds managed by local GP or consultant. Patients = medical/assessment, rehab, booked/emergency respite. | Identify factors that contribute to length of stay in community hospitals by reviewing 202 patient records. | Number of factors associated with increasing length of stay - age, gender, functional ability, risk of malnutrition, discharge arrangements etc. Authors make several recommendations to minimise excessive length of stay including discharge preparation on admission, multidisciplinary communication etc, |
| [18] | Carson, M. and Close, J.A team approach to the audit of nutritional care in community hospitals | 1996 | England | Audit | Questionnaire | 11 community hospitals in Norwich. | To examine nutritional care received by patients in community hospitals. Methods including dietetic assessment of menus and food eaten, consideration of menu cycle, choice and content, survey of nutritional knowledge amongst nurses and survey of patient satisfaction | Few conclusions made. Authors state that an 'action plan and re-audit tool' have still to be written. Suggestions based on results include an increase in dietetic sessions, the introduction of a nutrition assessment tool (to take weights/portions etc. of food into consideration) and further training and education for catering and nursing staff. |
| [19] | Cavanagh, A. J. Community hospitals | 1995 | UK | Commentary / Non-systematic review | Literature / expert | UK community hospitals in general. | N/A. | Report mainly provides a descriptive account of factors relating to rural health and health service provision by GPs. Covers areas like urban Vs rural health differences, needs of rural residents, GP recruitment, workload and education. Chapter by Cavanagh describes the role of CHs in rural areas - discusses politics, history, access and distance, functions, costs, workloads for GPs, recruitments, impact of secondary care, audit and operational procedures. |
| [20] | Cavenagh, A. J., Phillips, K. M., Sheridan, B., and Williams, E. M.Contribution of isolated general practitioner maternity units | 1984 | UK | Cross-sectional survey | Questionnaire | 131 isolated GP maternity units in England and Wales were identified. 116 units responded  Average bed capacity was 12 per unit (range 1 to 31). Average of 16 miles from nearest district hospital (range 1 to 57 miles). 35% of units had the potential to be cut-off by bad weather. 87% of units were integrated with community midwife service. | To assess the activities of isolated GP maternity hospitals and assess perinatal mortality. | Delivery in isolated GP maternity units is safe. Increasing the operative role of GP obstetricians may improve results. |
| [21] | Church, J. and Seamark, D.A survey of surgical activity in UK community hospitals | 2002 | UK | Survey | Questionnaire | All 471 CHs in UK (of which 79 provide in-patient and day-care surgery). Total of 18,579 beds (of which 752 surgical) | Survey of surgical activity in UK CHs to determine (mainly by questionnaire) the proportion providing surgical services, staffing, cases, type of anaesthetic, type of surgical procedure and equipment used. | 17% of CHs perform surgery with approx. 70,000 cases/year. GA used for 45%. Range of procedures wide. Equipment used included laparoscopes, arthoscopes and operating microscopes. Authors conclude that expansion of surgical activity within CHs is feasible. |
| [22] | Coast, J., Inglis, A., and Frankel, S. Alternatives to hospital care: what are they and who should decide? | 1996 | England | Observational (retrospective cohort) | Patient data, panel of clinicians re-evaluate | Acute admissions to one DGH in rural setting in England | To assess the proportion of patients who could have been cared for in an alternative setting to the DGH in retrospect | 5-14% of patients estimated to be suitable for alternative treatment (mainly urgent out patient clinic or CH bed). |
| [23] | Coast, J., Inglis, A., Morgan, K., Gray, S., Kammerling, M., and Frankel, S. The hospital admissions study in England: are there alternatives to emergency hospital admission? | 1995 | England | Observational (Economic ) | Cohort (retrospective) | Acute hospital (no details of hospital or location, authors address given as Bristol).  Examined 701 acute admissions to acute hospital over period of 6 months- November 1992 to May 1993. Study selected admissions on every 4th day. Data from 677 admissions analysed. 68% admissions were to general medicine, 32% were to geriatrics. 125 patients classified as suitable for alternative forms of care by researcher using validated tool- the 115 patients who had been admitted via GP or the A & E department were further classified by a panel of 7 GPs as suitable or not for alternative care. 12 alternative forms of care were offered. | To assess the potential for substituting alternative forms of care for admission to an acute hospital in particular groups of patients. | The authors concluded that there is the potential for treating a proportion of patients in lower intensity alternatives to the acute hospital. However, this would result in few resource savings. . |
| [24] | Cook, P. J. and Porter, L. Community hospitals and district general hospital medical bed use by elderly people: A study of 342 general practitioner beds in Oxfordshire | 1998 | England | Observational | Hospital data | Oxfordshire Health Authority (population 510,000). 11 community hospitals (9 - 62 beds) and 2 DGHs. 90% of beds in CHs used by elderly patients. | To examine the association between GP access to CHs and distance from DGH with DGH medical bed use by elderly people. | There is a small effect of DGH bed use associated with access to CHs which 'almost disappears' when distance from DGH is controlled for in the regression analysis. Bed use falls with distance from the practice surgery to both CH and DGH. |
| [25] | Cormie, P. Palliative care in community hospitals in the Borders. | 1999 | Scotland | Cross-sectional survey | Questionnaire Hospital data | Scottish Borders, rural area population 105, 700. No hospice. Palliative care service based at Borders General Hospitals. Most patients admitted for palliative care are under the care of their GP in one of seven community hospitals. | To examine current palliative are provision in the Borders with respect to 7 predefined areas (quality, facilities, staffing, communication, education, audit and evaluation and development planning). And to compare the service with the recommendations of the Scottish Partnership Agency. | Makes 12 recommendations that need to be implemented if the service is to meet the recommendations of the Scottish Partnership Agency. |
| [26] | Dick, R. W. Inquest on QUEST | 1996 | Scotland | Audit | Patient data | 31 wards audited within Community Health Services Unit of Grampian Health Board using general elderly services Quest. Some had repeated audits | Examines and assesses impact of Quest (Audit tool) on service provision within group of small local community hospitals; | Author states difficult to draw any firm conclusions from analysis of audit using methods described in study. Authors of Quest audit identified key areas in overall delivery of a particular aspect of health care provision |
| [27] | Donald, I. P., Jay, T., Linsell, J., and Foy, C. Defining the appropriate use of community hospital beds | 2001 | England | Observational | Cohort (prospective) | Nine community hospitals in Gloucestershire with total of 283 beds. 3862 admissions in 1997. | To develop a protocol so that the appropriateness of admissions to community hospitals can be assessed. | 5951 community hospital bed days assessed. Using protocol, 82% of admissions satisfied criterion. 15% of admissions might have been avoidable if alternative existed. |
| [28] | Ebmeier, K. P., et al. Continuing care of the demented elderly in Inverurie | 1988 | Scotland | Descriptive | N/A | Peripheral psycho-geriatric unit set up at Inverurie cottage hospital, Aberdeenshire in 1982 to meet increasing demand for long-term care of the demented elderly in the area. Total catchment population was (1986) 43, 000. 2,700 (6%) > 65 years; 2400 (5.5%) > 75 years. Hospital has 2x 24-bed geriatric wards and 1x 10 -bed Maternity unit. Staffed by nurses (mostly generally trained) plus Inverurie GP practice providing 24-hour cover, occupational therapist, chiropodist, social worker. Consultant psychiatrist plus registrar assess patients in community. | To describe the psycho-geriatric unit set up at Inverurie cottage hospital. | Advantages of the psycho-geriatric unit and current organisation include ease of access and an identifiable resource for relatives and GPs, an adequate and flexible response to crisis, respite admissions and day care can be quickly provided and are more acceptable, high staff moral from involvement in regular meetings. Potential disadvantages have not been significant. |
| [29] | Edwards, N. Models of perfection... small general hospitals will have to adapt and change | 1998 | UK | Expert opinion | N/A | UK community hospitals in general. | N/A. | Author identifies models that could be used in order that 'small hospitals' (serving populations of less than 150,000) can adapt to changing demands - mainly related to specialisation and costs of care. Proposes enhanced ambulatory care, elective inpatient surgery, urgent GP consultations and outpatient services. A & E and paediatrics should be delivered by DGHs. |
| [30] | Garrett, T., House, W., and Lowe, S. W. Outcome of women booked into an isolated general practice maternity unit over eight years | 1987 | England | Observational | Retrospective cohort | Keynsham maternity unit, 6 miles (15 mins by ambulance) from specialists units at Bath & Bristol. Travel time prolonged by traffic. Experienced midwives staff 20 beds, 8 used for postnatal care of women transferred from specialist units. 10 GPs use unit for intrapartum obstetrics. No facilities for Caesarean section, no specialist anaesthetic or paediatric cover on site but neonatal resuscitation facilities available. GPs attend deliveries. Bookings mare for low-risk women. | Outcome of women booked into an isolated GP maternity unit over 8 years (also includes data of transferred and changed booking cases and how this influences outcome of group as a whole - this differs from study id 321) | Authors conclude there is now sufficient information concerning risk of transfer and change of booking from studies of isolated GP maternity units for pregnant women at low risk to make informed choice of place of delivery |
| [31] | George, J. Community hospitals...and district general hospitals [7] | 1999 | UK | Expert opinion | N/A | N/A | N/A. | Two letters.  1) Refers to a study which conflicts with findings of 17429 by showing a decline in DGH bed use with GP access to CH. Varian et al. (1996) Audit of a community hospital. Audit trends, 4, 88-90. 2) Author - Knox, J. Mentions cost comparison between CHs and DGHs. Concludes that cost of ward is same in both settings and as access to CHs increases overall bed use, additional expenditure occurs. |
| [32] | Grant J, Ramsay A, Bain J. Community hospitals and general practice; extended attachments for medial students. | 1997 | Scotland | Descriptive (Questionnaire) | Student evaluation questionnaire | Ten general practices each linked to a community hospital in either Tayside or Grampian; 10 fourth-year undergraduate medical students from Dundee Medical School, each attached to one of the practices. | To evaluate the educational effectiveness of prolonged clinical attachments for medical undergraduates in community hospital-based general practice. | The authors concluded that prolonged attachments in community hospital-based general practice for medical undergraduates was educationally valid and popular with both students and tutors. The extension of this model on a wider basis has resource issues that have to be addressed. |
| [33] | Grant, J. A. and Dowell, J.A qualitative study of why general practitioners admit to community hospitals | 2002 | Scotland | Qualitative | Interviews | Five community hospitals in Tayside. Ten GP practices with admitting rights. District General Hospital 14-30 miles away. | Identify factors associated with GPs' decision to admit patients to community hospital by conducting in-depth interviews with 43 GPs (purposive sample) | Number of factors identified. Primary influences = 1) Context at time - hospital capacity, staffing numbers etc. 2) Feelings at time - type of admission, 'comfort' level of GP 3) patient preference |
| [34] | Grant, J. A. Community hospitals--time to come off the fence.[see comment] | 1989 | Scotland | Expert opinion (commentary / non systematic review) | Literature / expert | UK community hospitals in general. | An argument for GP beds within modern General Practice | General practice needs to reconsider attitude to intermediate care |
| [35] | Grant, J. A. Contribution of general practitioner hospitals in Scotland | 1984 | Scotland | Survey | Questionnaire | 70 Community Hospitals in Scotland surveyed. 64 responded. All beds were designated as short stay, medical, long stay, surgical or obstetric. Information requested for 1980. | To assess the contribution of GP hospitals to the overall hospital workload. | The contribution of GP hospitals to the health care of communities cannot and should not be underestimated. |
| [36] | Griffiths, P. D., Edwards, M. E., Forbes, A., Harris, R. L., and Ritchie, G. Effectiveness of intermediate care in nursing-led in-patient units (protocol and full report). | 2004 | UK | Systematic review | Literature | 11 studies (3 conducted in US, remainder in UK). 10 are controlled trials involving 1896 patients. | Compare nursing led in-patient units (where nurses have the authority to admit and discharge) with usual patient care managed by doctor. Comparisons based on 'effectiveness' in preparing patients for discharge. | Patients discharged from nursing led units are more independent (in terms of functional status) and experience greater well being. There are also fewer discharges to institutional care and length of stay in nursing led units is significantly longer than usual patient care units. No differences in terms of mortality. Costs of care in UK studies were higher. |
| [37] | Griffiths, R. A. and Wilcock, G. K. Geriatric medicine in two community hospitals - The Oxford experience | 1981 | England | Descriptive | N/A | Oxford geriatric units described - set in specially designed community hospital and compared with GP care at a nearby CH | None. | The modus operandi of two community hospitals developed to care for the elderly are described. The system provides for continuing care of the elderly near their own homes; support for relatives; a reduced burden on acute beds in the general hospital; more direct involvement of GPs in resolving medico-social problems; and a reduction in workload on central consultant services. |
| [38] | Hall, R., Ramaiah, R. S., and Jones, D. T. General practitioner hospitals: coming or going? | 1984 | Wales | Descriptive | Patient data | Clwyd, Wales. Population aged 65 years and over of 62,000. Area has networks of GP hospitals to complement two district hospitals. | To assess the contribution made by GP hospitals in the short term care of the elderly between 1978 and 1982 by comparing annual discharge/ deaths between GP hospitals and geriatric units. | GP hospitals provided care for an increasing number of older people between 1978 and 1982 |
| [39] | Halpin, S. F., Yeoman, L., and Dundas, D. D. Radiographic examination of the lumbar spine in a community hospital: an audit of current practice. | 1991 | England | Survey | Questionnaire | London Community Hospital | To assess GPs decisions to request lumbar spine radiographs according to guidelines of Royal College of Radiologists | Authors conclude need to inform doctors of efficacy of radiological examinations; awareness of college's guidelines among GPs should be actively promoted by radiologists |
| [40] | Hamilton, W. and Round, A. Urban community hospitals | 1995 | England | Survey | Questionnaire | All GPs in Exeter surveyed. 76 questionnaires sent out, 72 returned (95%); | To determine GPs opinions on urban community hospitals | Author concludes high response rate reflects relevance of topic, but only 49% of GPs in favour of an urban community hospital; Proposed positive primary health care reasons for their support eg respite care; An urban community hospital would provide services not now available rather than an alternative to district general hospital admission. |
| [41] | Harris, G. The context and content of community hospital admissions | 1986 | England | Cross-sectional survey | Hospital patient data | Community Hospital situated in town of 3,000 inhabitants, serves 7,000 people in surrounding villages. Has 34 beds and outpatient, casualty and physiotherapy depts. Day centre provides rehab and support for older patients and their carers. No surgical or obstetric beds; admissions due to acute problems; Medical staff comprise 9 GPs; majority of admissions from one practice. | Hospital admissions from a general practice studied for one year. Assessed needs of patients for medical, nursing and social services | Authors concluded social, nursing and medical factors determined admission and discharge. Social factors found to be increasingly important as the age of patients increases while medical factors become less important |
| [42] | Hawkins, A. Triage in rural hospitals |  |  | Paper not available |  |  |  |  |
| [43] | Haynes, R. M. and Bentham, C. G. Community hospitals and rural accessibility | 1979 | England | Case-study | Questionnaire Interviews | King's Lynn Health District - population 168,000. 12 hospitals in area with a total of 830 beds - including a General Hospital | To explore the feasibility of implementing a community hospital policy in a rural area by conducting a case-study in the King's Lynn Health District. Covers factors like accessibility, staff attitudes, costs of service, costs to patients and patient satisfaction. | Several - see last chapter. Those which may be applicable to other settings include: Improved accessibility increases use of outpatient services and number of visits to inpatients. Some patients benefit socially and psychologically from smallness and accessibility of CHs CHs will compete for staff with other hospitals Staff generally agree that care should be GP led and functions of CHs should include geriatrics, medical and surgery care and convalescence. |
| [44] | Haynes, R., Gale, S., Mugford, M., and Davies, P. Cataract surgery in a community hospital outreach clinic: patients' costs and satisfaction | 2001 | England | Controlled trial - not randomised (Survey) (Qualitative) | Questionnaire interviews | Cataract day surgery service in Norwich area provided at 1) District General Hospital and 2) outreach clinic at community hospital 40 KM away. Community hospital = 58 beds. | To compare 201 patients who attended DGH with 198 patients who attended outreach clinic. Both groups of patients attended cataract day surgery service. Questionnaires and interviews determined personal information, travel costs, levels of satisfaction, surgery outcomes etc. | Response rate high (85% and 92%). Few differences found except: some aspects of good health prior to surgery significantly better amongst patients attending DGH, costs to patients for attending outreach clinic less, significantly higher levels of satisfaction amongst patients attending outreach clinic. |
| [45] | Heard-Dimyan, J. Issue of emergency hormonal contraception through a casualty department in a community hospital | 1999 | Wales | Audit | Patient data Questionnaire | Casualty department of Brecon War Memorial Hospital in Powys, Mid Wales. Serves a market / garrison town, population 15,000, including army families. Hospital staffing and size not stated. Casualty department had two nurse prescribers. | Assess practicality and uptake of emergency hormonal contraception prescription from a community hospital. | Acceptable to attenders. Biggest uptake in younger age groups. Saved travel, and demonstrated that nurse prescribers could offer a service. |
| [46] | Hegney, D., McCarthy, A., and Pearson, A. Professional issue. Effects of size of health service on scope of rural nursing practice | 1999 | Australia | Audit | Questionnaire | Rural hospitals in Australia - not always clear if general hospital or Community hospital but covers full range of sizes | Analyse the activities of rural hospital nurses | Size of the unit is a major determinant in the skill mix and role of nurses |
| [47] | Henderson, L. R. and Scott, A. The costs of caring for stroke patients in a GP-led community hospital: an application of programme budgeting and marginal analysis | 2001 | Scotland | Economic | GP medical records, NHS cost data | Data collected for all patients with diagnosis of stroke in 19945 and 1995/6 in Nairn and Ardersier GP practices. Highland Health Board. List size 13,500 patients, 2 GP practices with 10 GP principals, semi-rural area 15 miles from Inverness.  Examined resource implications of introduction of GP-led integrated care for stroke patients in the Nairn and Ardersier Total Purchasing pilot site. Care included care in Nairn and County Community Hospital and direct referral by GPs for CT scan. | To use the Programme Budgeting and Analysis and Marginal Analysis (PBMA) pragmatic economic evaluation framework to model probable changes in resource use resulting from the introduction of a GP led integrated care for stroke patients in the Nairn and Ardersier Total Purchasing Pilot Site. | The authors concluded that a policy of early discharge or direct admission to the community hospital may reduce total annual costs for treating stroke patients from $277,702 (£183, 000) to, at most, $112,295 (£74,000) per annum. Current evidence suggests that there is no associated change in health outcomes but further research into heath outcomes and quality of life is required before widely adopting this policy. |
| [48] | Higgins, J. Community hospitals. Cottage industry | 1994 | England | Expert opinion | N/A | Community hospitals in 1990s | Analysis of impact of policy on community hospitals | Changes in policy allow innovation, diversity and local responsiveness |
| [49] | Higgs, R. Example of intermediate care: the new Lambeth Community Care Centre | 1985 | England | Descriptive | N/A | New Lambeth Community Care Centre opened in 1985 in South London. Provides intermediate care in the community. Has 20 adult beds and 35 day places. Inpatient facilities for postoperaitve care, terminally ill, patients needing acute but not specialised medical care, and respite relief for carers of disabled patients. Day unit for similar population plus patients with impaired mobility and ability to undertake activities of daily living.  Care provided by GPs | To describe the facilities to be offered at the new New Lambeth Community Care Centre. | Descriptive only- no true conclusions. States the centre is one response to the challenge of intermediate care and that there is a need to continually assess the quality and direction of its activities. offers |
| [50] | Hindle, H., Norheim, J. K., and Renger, R Rural Alberta thrombolysis study. Survey of practice patterns for managing acute myocardial infarction | 1995 | Canada | Cross-sectional survey | Questionnaire | Thrombolysis in rural hospital with transfer to larger unit thereafter. Initial assessment by GP and thrombolysis administered at GP led hospitals | To describe the current practice for managing MI and examine potential barriers to thrombolysis in community hospitals | Thrombolysis is standard practice. Where barriers exist, it is seen to be due to a lack of skilled nurses |
| [51] | Hine, C., Wood, V. A., Taylor, S. and Charny, M. Do community hospitals reduce the use of district general hospital inpatient beds? | 1996 | England | Observational | retrospective cohort | Part of review of community hospitals in Bath Health District in 1992. Facilities include 900 bed DGH plus care of the elderly hospital in Bath, 18 community hospitals in small towns with GP input and three geriatric hospitals.  Study compared bed utilisation rates in DGH and community hospitals for 15 GP practices in Bath city with no access to community hospitals with 32 GP practices in small towns and rural areas with access to community hospitals. | To compare utilisation rates for inpatient beds for communities with and without access to community hospitals in Bath Health District. | Community hospitals provide one option for accessible health care and because of this an evaluation of the costs and benefits are required. Some evidence was found suggesting that increased efficiency could result in savings. |
| [52] | ISD Community hospitals information project: final report. | 2001 | Scotland | Descriptive (Qualitative) | Interviews | 2 stage Project 1. Interviews with medical, nursing and management staff at 26 (33%) Community Hospitals in Scotland to determine the requirements for routine data collection in Community Hospitals.  2. Data set developed from results of stage 1 and paper version of proposed dataset piloted in 4 Community Hospitals. | To describe the extent to which current routine data collection methods meets information requirements for Community Hospitals, to develop a revised dataset, to pilot the proposed data set and to evaluate the revised dataset. | It is feasible and highly desirable to collect clinically useful information at little additional cost and to provide relevant feedback, including useful patient summary screens representing the patient stay in hospital. |
| [53] | James, A. M. Closing rural hospitals in Saskatchewan: on the road to wellness? | 1999 | Canada | Commentary / Non-systematic review | Literature / expert | Reforms to the Canadian health system have resulted in closure of rural hospitals in Saskatchewan and their conversion to wellness centres. Reforms have resulted in provinces moving to global hospital funding. | To review the importance of local hospitals to communities in the light of closure of rural hospitals resulting from reforms in the Canadian Health system. | There is a need to consider the impact of closure of rural hospitals in terms of health irrespective of the medical impacts. |
| [54] | Jarman, B. Community hospitals: a desirable option? | 1995 | England | Expert opinion | N/A | Inner city community hospitals | Discusses the development of inner city community hospitals | Development dependent on resources, commitment of professionals and patient preferences |
| [55] | Jeavons, R. and Taylor, P. Review of community hospitals |  |  |  |  |  |  |  |
| [56] | Jeffrey, D. Education in palliative care: a qualitative evaluation of the present state and the needs of general practitioners and community nurses | 1994 | England | Survey | Questionnaire | Worcester Health District | To assess needs of GPs, community hospital nurses and CNs for education in palliative care | Need to improve training. Time and resources required |
| [57] | Johnson, D. B. Audit of surgical practice in a community hospital | 1984 | Wales | Audit | Patient data | Community hospital of 40 beds in rural mid-Wales run by practice of 8 GPs. Overall catchment 18,000 to 20,000; Each GP has list of av 1700 patients and a speciality interest: 1 general surgeon; one obstetrician/gynaecologist; one ear, nose & throat surgeon, 2 anaesthetists (one with FFARCS), one general physician, one paediatrician, one partner with interest in orthopaedics; Hospital provides general medical and surgical care to particular level then patients referred to district general hospital 20 miles away; an obstetric service (with facilities for caesarean section, foetal monitoring); casualty service; facilities for outpatients, radiology & physiotherapy.; Hospital performs limited range of straightforward surgery on fit patients; some emergency procedures undertaken. | Audit of surgical practice in a community hospital, from medical and patient's point of view to attempt to reach conclusions regarding contribution, quality and safety. | Authors conclude that high quality of surgery may be achieved with safety and low rates of complications; also applied to results of retrospective analysis of certain aspects of surgery. Community hospital surgery is convenient for patient, provides continuity of care by GP, waiting list times short; Surgical facilities can form integral part of comprehensive service and can lighten caseload for minor surgery at district general hospital, but close liaison between hospitals essential. |
| [58] | Jones, R. and Tucker, H. The role of community hospitals | 1988 | England | Commentary/ Non-systematic review (survey) | Literature Questionnaire survey | English community hospitals | Current and future role of community hospitals | Community hospitals need to be measured and compared to DGH's in order to define appropriate use |
| [59] | Jones, R. General practitioner beds in Finland - lessons for the UK? | 1987 | Finland | Descriptive | N/A | Finland, GP inpatient hospitals, of similar scale to UK. Almost every "health station" - 10-15,000 people has in patient GP run beds and rising. Now accounts for 20% of all inpatient beds. | To explore the Finnish model in order to identify lessons for the UK | While focus has been on primary care and model is of use - it is flawed. Health stations do not ensure GP continuity of care as there is no GP registration system. Visits to Health station are akin to UK outpatient clinics - seeing a different Dr every time. The stations have their own staff and hospital Dr and are much more high tech than UK cottage hospitals. The model does however demonstrate that it is possible to integrate planning for GP beds into the management structure. The role for specialist outpatient support is important along with the inpatient beds |
| [60] | Jones, R. Do general practitioner hospitals extend primary care? | 1986 | UK | Expert opinion | N/A | UK community hospitals in general. | N/A. | Author addresses paradox when some community hospitals are faced with closure while others are opening. Notes the absence of policy regarding use and development of CHs. Talks about the contribution CHs can have in terms of acute, terminal and elderly care. Also mentions continuity of care, cost-effectiveness, decentralisation and extension of GP skills. Very pro CH. |
| [61] | Kerrin, D. and Jones, R. Community hospitals. | 1989 | England | Survey | Questionnaire | 145 acute medical admissions to Southampton General Hospital; | To determine views about feasibility of GPs caring for patients in a community hospital setting (exact community hospital was hypothetical) | Authors conclude results suggest that substantial number of patients admitted to district hospital could be cared for in GP units although is a lack of consensus between health care professionals on this. Studies measuring cost effectiveness of community hospitals needed. |
| [62] | Latimer, J. Nursing in a different way | 1987 | England | Descriptive | N/A | New GP community hospital in inner London. No detail of bed numbers or what type of patients were admitted. 48 GPs with no experience of community hospital Nurses control beds and decide who to admit and when to discharge. | Describes positive aspects and stresses of setting up and running a new community hospital from a nursing perspective. | Appears to conclude that the opening of the community hospital meant nurses had to redefine their role. |
| [63] | Legge, A. Nurse-led hospital service takes on GPs' night calls | 1998 | England | Descriptive | N/A | Community hospital in Swanage, small coastal town near Bournemouth. Has nurse-led minor injuries unit, 24 in-patient beds. Nurses extended activities and took over the GPs out of hours calls in 1995. | Describes take over of GPs out of hours calls by nursing staff at community hospital | No conclusions- just description |
| [64] | Lemaire, E. D., Boudrias, Y., and Greene, G. Low-bandwidth, Internet-based videoconferencing for physical rehabilitation consultations | 2001 | Canada | Descriptive | N/A | Videoconferencing set up linking 8 community hospitals in Ontario province, Canada with one specialised multidisciplinary outreach physical rehabilitation hospital in Ottawa. | To describe the use of videoconferencing for physical rehabilitation consultations. | The study supports the use of videoconferencing for many remote physical rehabilitation consultations. |
| [65] | Licence, K., Crichton, C., and Stark, C. Changes in the pattern of work in a rural community hospital 1987-1997 | 1999 | Scotland | Observational | Hospital patient data | Island community hospital | To examine trends in clinical activity in a rural hospital and assess changes in care received by in-patients | Change in the use of the hospital: increase in activity and clinical spectrum of admissions -role of hospital is the provision of intermediate care |
| [66] | Liddell, R., Grant, J., and Rawles, J. The management of suspected myocardial infarction by Scottish general practitioners with access to community hospital beds.[see comment] | 1990 | Scotland | Survey | Questionnaire | 20 community hospitals serving population of 149,000;  Located in market towns mean 38 miles from district general hospital (range 4 to 120 miles); Size ranged from 6 to 43 beds (mean 16); 3 hospitals on islands where nearest hospital via sea ferry or air ambulance).   All hospitals in survey equipped with defibrillators and ECG; cardiac arrest trolley or box; 7 hospitals did not have ECG and 10 hospitals no formal tuition in cardiopulmonary resuscitations given to nursing staff within last 3 yrs. In none of hospitals was defibrillation officially recognised as a nursing duty. | Management of suspected myocardial infarction by Scottish GPs with access to community hospital beds | Authors concluded in rural areas of Scotland an acceptable standard of care for patients with acute myocardial infarction (including administration of thrombolytic therapy) could be provided rapidly by GPs working in Community Hospitals |
| [67] | Light, D. and Dixon, M.A new way through | 2000 | UK | Expert opinion | N/A | Not localised. | To discuss collaborating contracting as a tool for developing effective intermediate care. | The new NHS needs to start collaborating contracting pilots for intermediate care as soon as possible. |
| [68] | Liu, L., Hader, J., Brossart, B., White, R., and Lewis, S. Impact of rural hospital closures in Saskatchewan, Canada | 2001 | Canada | Survey | Questionnaire, Interview, hospital data | 52 rural hospitals were closed in Saskatchewan in 1993 and most were converted to primary care centres (most of closed hospitals were funded < 8 beds). Most of the hospitals were located in towns with < 500 people and about 50% had integrated long-term care facilities. Many of the replacement primary care centres (wellness centres) are staffed 5 days a week with full time nurses and visits by physicians. | To assess the impact of hospital closures on patterns of hospital use, health status, rural residents' perceptions, and response of communities to closure. | Good rural health care does not depend on the presence of a very small hospital. It requires creative approaches to the provisions of primary care, good emergency services, and good communication with the public on the intent and outcomes of change. |
| [69] | Llewellyn, J., Evans, N., and Walsh, H. Service provision. The role of the community hospital in the care of dying people | 1999 | UK | Commentary / Non-systematic review | Literature / expert | Says is discussed with reference to Blaenau Gwent, South Wales community hospitals but presents no evidence from those community hospitals | To discuss the role of the community hospital in the care of people dying from cancer. | Community hospitals may be an ideal setting in which to care for terminally ill people who do not require specialised hospice beds. Further research is required to fully establish and evaluate the role of community hospitals in the provision of terminal care. |
| [70] | Lloyd-Williams, M. Survey of palliative care in a general practitioner unit | 1996 | UK | Cross-sectional survey | Case note review, | Two bed continuing care unit attached to a community hospital. Nurse and GP staffing mentioned. Two bed unit originally funded by the Cancer Relief Macmillan Fund. | To determine reasons for admission to the unit; look at drug use in the terminal phase of illness, and examine GPs role in provision of palliative and terminal care in a community hospital. | All practices had used the unit in an 18 month study period, plus a few out of area admissions. Evidence that 'as required' medications not always prescribed in advance. Limited palliative procedures undertaken: mainly terminal care. Limited recording of information on prognosis and communication. |
| [71] | Lowe, S. W., House, W., and Garrett, T. Comparison of outcome of low-risk labour in an isolated general practice maternity unit and a specialist maternity hospital | 1987 | England | Observational | Retrospective Hospital data | Keynsham maternity unit, 6 miles from Bristol maternity hospital; GP unit had 20 beds, 9 experienced midwives, used by 30 local GP for antenatal, intrapartum and postpartum care. Approx 140 women per yr booked for delivery unit. Transfers in labour either to Bristol maternity hospital or Royal United hospital Bath, also 6 miles away | Outcome of labour of low-risk pregnancies at isolated GP maternity unit compared with comparable low-risk pregnancies at specialist maternity unit | Authors conclude results suggest that where considerations for selection of low-risk pregnancy permit, GP maternity unit can provide distinctive style of intrapartum care with minimum intervention |
| [72] | Lyon, A. and Love, D. R. Terminal care: the role of the general practitioner hospital | 1984 | Scotland | Cross-sectional survey | Retrospective questionnaire | Peebles and surrounding rural area.  26 bed GP hospital looked after by 4 GPs caring for 9,000 patients (6,000 resided in town of Peebles).  218 patients, including 62 cancer patients, received terminal are over the 2 -year period surveyed. | To assess the role of the GP hospital in providing terminal care. | GPs provided terminal care for the majority (68%) of their cancer patients either in the GP hospital or at home. It appeared that home-based care was less common where there was the alternative of a GP hospital. |
| [73] | Mabrook, A. F. and Dale, B. Can nurse practitioners offer a quality service? An evaluation of a year's work of a nurse led minor injury unit.[see comment] | 1998 | UK | Other experimental | No comparator  Hospital data Questionnaire | Minor injuries service provided by emergency nurse practitioners acting in extended role. Worked to protocols with support of larger medically staffed A&E by patient transfer | Evaluate the activities of a nurse led injuries unit based in a community hospital | With planning, supervision and training nurses can provide an effective service |
| [74] | Macduff, C., West, B. J. M., and Lawton, S. An evaluation of the impact of developing nurse-led treatments for minor injuries in community hospital casualty units... including commentary by Dolan B | 2000 | Scotland | Cross-sectional survey |  | Nurse-led minor injuries units at nine community hospital casualty units in Grampian region of Scotland. | To outline a nurse-led minor injury service at community hospitals and report the main findings on its evaluation and the implications. | The development had a positive impact on professionals and waiting times appear to have been reduced. The educational course for participating nurses was an important factor in the developments success. |
| [75] | McConnon, J. K. The Canadian community hospital | 1987 | Canada | Expert opinion | Literature / expert | 525-bed community hospital, near Toronto, 60 specialists and 120 GPs, serving community of approx 250,000. No "residents" and their work done by GPs who run A&E, maternity, minor surgical and gynaecological procedures. | Compares Community hospital setup in Canada with underlying assumptions in UK & USA systems | Compares differences in career structures between Canada, US & UK. No particular conclusions reached by author. |
| [76] | McCormack, B. Community hospital-issues for older people | 1998 | England | Expert opinion | N/A | Role of community hospitals for older people | Discusses the role of community hospitals in the provision of care of older people | Examines why community hospitals are under threat of closure and considers method by which nurses are prepared to practice |
| [77] | McCormack, B. The developing role of community hospitals: an essential part of a quality service | 1993 | England | Expert opinion (commentary / non systematic review) | Literature / expert | UK community hospitals using Oxfordshire as an example | Considers the interface between primary and secondary care and explores the future of community hospitals | A community hospital can contribute to the overall pattern of health care and offer an environment to develop models of health care |
| [78] | McKinlay, W. J. D. Why we need community hospitals for the elderly. | 1991 | UK | Expert opinion | N/A | UK community hospitals in general. | N/A. | Discusses the advantages of community hospitals with particular reference to care of the elderly and author's own local unit. Believes CHs can be cost-effective. CHs provide continuity of care in a 'homely' environment and locations benefit both patients and carers. CHs have the capability to provide medical procedures not available at home |
| [79] | Meads, G. Rediscovering community hospitals | 2001 | UK | Expert opinion | N/A | UK community hospitals in general. | N/A. | Expansion in number of community hospitals and allocation of extra funding (NHS Plan 2000) offers 'exciting' prospects for primary care. Future will lead to transformations based on 1) rediscovery of primary care as providers 2) addressing needs of handicapped, disabled and disadvantaged groups 3) political devolution 4) decentralisation of NHS |
| [80] | North, N. T., Hall, D. J., and Kearns, W. E. First year of an inner city general practitioner community hospital | 1984 | England | Descriptive | N/A | Inner city community hospital in Bayswater. 24 beds. 48 GPs have access. Over 12 months, 316 admissions. Staffing = 9 nurses, 12 auxiliaries, physiotherapist, occupational therapist and social worker. Visiting professionals = speech therapist, dietician and dentist. | Describe the development and operation of the CH in its first year. | CH provides continuity of care. Procedures such as home assessments prior to admission and phased care schemes, which allow carers to input, help alleviate distress. GPs are able to use range of skills and job satisfaction amongst GPs is improved. |
| [81] | Palombo, A., Ferguson, J., Rowlands, A., Pedley, D., and Fraser, S. An evaluation of a telemedicine fracture review clinic | 2003 | Scotland | Audit | Patient data | See ref 13 for full details of telemedicine project- limited information given in this report. Telemedicine project linking A & E department in Aberdeen with minor injury units at community hospitals. This report is about the telemedicine fracture review clinic-Reports on the number of patients seen in telemedicine review clinic, number seen in Aberdeen who had no nearly telemedicine facility and the number seen in Aberdeen who had a nearby telemedicine facility.  Telemedicine clinic runs at same time as fracture review clinic in Aberdeen | To evaluate a telemedicine fracture review clinic. | All patients were safely managed and normal guidelines were followed. There is scope for a significant expansion of the telemedicine fracture review clinic since 131 patients who attended Aberdeen had nearby telemedicine facilities. |
| [82] | Payne, S. and Ramaiah, R. S. The importance of general practitioner day hospitals | 1986 | Wales | Cross-sectional survey | Hospital data Questionnaire | 4 day hospitals in Clwyd (Chirk, Denbigh, Mold & Ruthin Community hospitals). Each is purpose built, has 15 places, serves population of 21,000, of which 2,500 >65yrs, and 1600 >75yrs. Do not exclude young chronic sick or younger disabled person. Referred patients do not need full complement of services provided by acute sector | Importance of GP day hospitals: review of patients attending 4 day hospitals in one week In Sept 85 | Authors feel that GP run day hospitals contribute significantly to community care of the elderly. Advantages: GP familiar with patients personal as well as medical circumstances; continuity of care throughout rehab as well as monitoring progress after discharge; Most patients live within accessible distances; management structure of nursing services encourages exchange of info between hospital and community staff. |
| [83] | Pedley, D., Ferguson, J., Palombo, A., and Richardson, J. Community coronary units: strategies to promote pre-hospital thrombolysis | 2002 | Scotland | Descriptive | N/A | Fourteen community hospitals in Grampian, which act as 'community coronary units' and have videoconferencing links to District General Hospital. Have also (recently?) received remote telemetry equipment in order to encourage pre-hospital thrombolysis | None | Authors suggest that existing videoconferencing and remote telemetry equipment can, in the future, be used by GPs to access specialist cardiologist support and advice for patients presenting with suspected MIs. |
| [84] | Pencheon, D. Intermediate care | 2002 | UK | Expert opinion | N/A | No specific location | Discusses need for evaluating intermediate care, rationale behind the development of intermediate care. | Conclusion appears to be along the lines that is not possible to judge whether intermediate care increases capacity and cost-effectiveness without evaluating this form of care. |
| [85] | Primrose, W. R. Community hospitals | 1998 | UK | Commentary / Non-systematic review | Literature / expert | UK community hospitals in general. | N/A. | Discusses the role of CHs with a particular focus on geriatrics. Provides definition, number, facilities, reasons for admission, GP access and role in rural areas. Also discusses lack of guidelines for care or studies of effectiveness, maintenance of skill levels, telemedicine, staffing issues, role of visiting specialists, educational opportunities for students and access/distance. |
| [86] | Ramaiah, S. Community hospitals in the new NHS.[see comment] | 1994 | England | Expert opinion | N/A | Community hospitals in 1990s | Analysis of impact of policy on community hospitals | Concern that the concept of neighbourhood care is under threat |
| [87] | Ritchie, L. D. and Robinson, K. Community hospitals: new wine in old bottles?[see comment] | 1998 | UK | Commentary / Non-systematic review | Literature / expert | UK community hospitals in general. | N/A. | Community hospitals continue to make significant contributions to patient care throughout UK. In current climate of resource constraints, way forward is for CH to establish place as part of a range of quality patient services. Systematic evidence and commitment needed to make that happen |
| [88] | Round, A., et al Six month outcomes after emergency admission of elderly patients to a community or a district general hospital | 2004 | England | Observational | Prospective cohort | One DGH and 5 community hospitals in Devon. No community hospital was available for catchment area of people treated at DGH.  Data collected for patients aged > 70 years, with acute illness (acute medical, emergency medical or geriatric medical) requiring hospital admission which could be treated in either setting. | To compare outcomes of emergency medical admissions in the elderly between community hospitals and DGH. | Emergency admissions to community hospitals and DGH had similar quality of life and mortality at 6 months. Community hospitals can be used as an alternative to DGH for a wide range of conditions requiring emergency admission. |
| [89] | Royal College of General Practitioners. Community hospitals: preparing for the future. | 1990 | UK | Expert group opinion (commentary/ non systematic review) | Literature / expert | UK community hospitals in general. | Look at literature related to volume of work, cost benefit and quality of care provided by 'GP hospitals'. Examine past/current research and audit activity. Make recommendations and suggest guidelines for patient care and management. | Describes GP hospitals in terms of background, roles, functions, standards of care, costs etc. and makes a number of recommendations including: 1) Greater recognition of CHs from health authorities. 2) Availiability of casualty, outpatient, day care facilities and high quality nursing in CHs. 3)Monitoring and auditing to become standard. 4) Recognition and promotion of multidisciplinary relationships. 5) Recognition of potential benefits to medical students. |
| [90] | Salmon, S., Brint, G., Marshall, D., and Bradley, A. Telemedicine use in two nurse-led minor injuries units | 2000 | Northern Ireland | Descriptive | N/A | Two nurse-led minor injuries units in community hospitals in Ards and Bangor, N Ireland. Units located 8 and 16 km from DGH. Units staffed by emergency nurse-practitioners. Units linked to specialist medical opinion at the A & E department at the DGH. Units saw 600 to 700 patients per month. | To describe the telemedicine use in two nurse-led minor injuries units. | Telemedicine worked well for patients with minor injuries. More detailed examination including a cost-effectiveness study and an assessment of users views is required. |
| [91] | Sanger, R. and Clyne, C. A. The surgical value of community hospitals: a closer look | Not on study (post 1988) | England | Cross-sectional survey | Hospital patient data  Questionnaire | Torbay District General Hospital, serves population of 250,000 plus tourists in summer months. Staffed by 5 surgical consultant firms each with 1 house office and one senior house officer and shares one associate specialist or registrar. Only acute general hospital in district with total of 128 surgical beds. Consultant firm has specialist interest in vascular surgery, vascular cases account for 50-60% of workload. 5 & half theatre sessions per week, including day theatre sessions on alternate weeks. Remainder of workload from one-fifth of shared general acute and elective admissions.   Newton Abbot Community Hospital - undefined population of Newton Abbot & Teignmouthareas. Situated 5 miles from District General Hosp, has 27 surgical beds and 1 operating theatre. Surgical beds shred among general surgery, gynaecology, ENT, orthopaedics & oral surgery. Staffed by resident senior house officer only, plus visiting consultant who visits on 4 of 5 week days. 1 full day theatre session available for routine surgical operations. 2 general surgical SHOs who alternate night cover with medical SHO. All varieties of elective operations carried out except major vascular cases or where post-op intensive care facilities required. Children >3yrs operated on for minor or int surgical procedures, but specialist paediatric nursing not immediately available. Beds shred with Local GPs also. No major Casualty or accident & emergency facilities, only emergency acute surgical admissions. | To assess and compare safety and efficiency of a surgical unit at a District General Hospital and a Community Hospital for elective cold surgery | Authors conclude community Hospital unit found to be satisfactory and more efficient for routine elective surgery as determined from parameters of study and patients themselves. |
| [92] | Scottish Partnership for Palliative Care. Palliative care in community hospitals: report of a working party of the Scottish Partnership Agency for Palliative and Cancer Care. | 1998 | Scotland | Expert group opinion | Literature / expert | Scottish community hospitals. | None. | Report summarises the role of community hospitals in providing palliative care and makes a number of recommendations to promote 'comprehensive planning' for palliative care services. Recommendations concerned with 1) collaborative working 2) criteria for admission 3) equipment/facilities 4) staffing - levels/training etc. 5) improving and maintaining quality and 6) financial implications |
| [93] | Seamark, D. A., Williams, S., Hall, M., Lawrence, C. J., and Gilbert, J. Dying from cancer in community hospitals or a hospice: closest lay carers' perceptions | 1998 | England | Qualitative | Interviews | Comparison of Community Hospital with Hospice (See component paper for further details). Setting 12 community hospitals in East Devon and a purpose-built hospice in city of Exeter. Hospice: 12 bed, nursing care provided by 13 whole-time equivalent trained nursing staff (RGN,SRN, or SEN), or 1.1 whole-time per bed; Community Hospital: 350 medical beds by 135 Whole time equivalent trained staff or 0.4 whole time equivalent per bed. | Comparison of perceptions of closest lay carers of cancer patients dying in community hospitals with perceptions of closest lay carers of cancer patients dying in consultant-led inpatient hospice | Authors conclude lay carers indicated great satisfaction with care given in hospice and less satisfaction with care given in community hospitals. Points out Community hospitals are non-specialist units, lower levels of trained staff. Improvements in terms of communication skills of doctors and nurses, specific training for nurses in palliative care & structured bereavement care could be made without necessarily increasing staff numbers. |
| [94] | Seamark, D. A., Williams, S., Hall, M., Lawrence, C. J., and Gilbert, J. Palliative terminal cancer care in community hospitals and a hospice: a comparative study | 1998 | England | Observational | Hospital data | Exeter, UK.  12 community hospitals (total of 350 beds available for patients with cancer) in 10 locations outside Exeter compared with 12-bed hospice on site of DGH in Exeter. | To compare a series of final admissions to community hospitals with final admissions to a hospice with respect to reasons for admission, symptoms, drug prescribing, interventions, and previous admissions. | There were differences in terminal care between the two settings. Differences in care between community hospitals and the hospice may reflect either different admission populations or differences in the way care was delivered. |
| [95] | Seamark, D., Moore, B., Tucker, H., Church, J., and Seamark, C. Community hospitals for the new millennium | 2001 | UK | Cross-sectional survey | Questionnaire | UK community hospitals in general. | To identify number of community hospitals in UK, distribution of these within England, Scotland, Wales and NI and to determine range and frequency of facilities offered. | Contrary to belief, number of hospitals has not declined. 471 in UK (85 in Scotland), 18,579 beds (2,963 in Scotland). Most common facilities = physiotherapy, occupational therapy, speech therapy, chiropody/podiatry. |
| [96] | Seamark, D., Seamark, C., and Lawrence, C. Contribution of community hospitals in East Devon to cancer workload. | 1997 | England | Observational | Patient data?? | Exeter Health District (population 315, 000) with 12 CHs - total of 500 beds | To examine the role of CHs in providing palliative and terminal care to cancer patients | Significantly more patients die at DGH and at home when GPs do not have access to CHs. CHs provided palliative care, investigations, active treatment and day-case surgery. Authors conclude that CHs have a role to play in continuing to provide care to cancer patients |
| [97] | Shacklady, J. and Browne, M. Community hospitals. Cornish hops | 1999 | England | Observational | Hospital patient data | Four community hospitals in East Cornwall. Total 89 beds. Adult inpatients transferred from 2 acute districts general hospitals and Royal Eye Infirmary serving population of 92,000. | To describe the success of a project set up Jan 1999 to Mar 1999 aimed at 1) facilitating earlier transfer from acute hospitals to community hospitals 2) 'successful' (not defined) discharge from community hospitals. Project involved appointment of two new members of staff - community resource facilitator (transfers) and specialist social worker (discharge) | Results were compared with same period in 1998. Total of 62 more patients transferred. Bed occupancy increased in 2/4 community hospitals. Length of stay increased in 3/4 community hospitals (authors believe due to hospitals receiving patients at earlier stage of care). Project "proved its worth to patients" and led to improvements in joint working. Authors believe longer trial needed to realise full benefits. Some issues over GP access to beds. |
| [98] | Shaw, C. D. and Collins, C. D. Health service accreditation: report of a pilot programme for community hospitals | 1995 | England | Descriptive | N/A | 57 community hospitals in South Western region of England were offered the accreditation programme. Hospitals had no resident medical staff and had < 50 beds and most beds were run by GPs. 43 hospitals accepted. | To describe the origin, operation and impact of a hospital accreditation programme for community hospitals. | The concept of accreditation may become an important factor in monitoring the quality of service in the new NHS |
| [99] | Shaw, C. D. General practitioner hospitals: coming or going? | 1984 | UK | Expert opinion | N/A | N/A | Seems to discuss the place of GP hospitals | There is little objective evidence that can be used to define the place of GP hospitals. Decisions must be made soon about the future of GP hospitals (1984). Health Authorities should carefully consider the economic and clinical potential of GP hospitals. |
| [100] | Simple solutions: clinical training for the whole staff: rural hospital puts all staff at the bedside | 2002 | USA | Descriptive | N/A | Brief news item about experience in training other staff to support nurses in rural hospital | N/A | N/A |
| [101] | Smith, K. Distance learning | Not stated | Scotland | Descriptive | N/A | Community hospitals in the north and east of Scotland linked via telemedicine with A & E consultants in Aberdeen. Enables GPs in community hospitals to obtain advice on managing minor trauma cases. | Describes a telemedicine project linking community hospitals with the A & E department in Aberdeen | No conclusions |
| [102] | Smith, M. The development of rehabilitation for older people in a community hospital. |  |  | Paper not available |  |  |  |  |
| [103] | Stark, C., Oliver, K., and Hopkins, P. Effect of general practitioner hospitals on district general hospital bed use in the Highlands of Scotland | 2000 | Scotland | Observational | Hospital patient data | All GP practices in Highland Health Board. Serving population of 215,069. 22% of populations covered by practices with access to GP beds | December 1995 - April 1997 = the use of DGH, geriatric medicine and total bed use examined amongst practices with and without access to GP beds | Access to GP beds was associated with 39.4% fewer bed days per 1000 patients for medical specialities. Also fewer bed days for surgical specialities, geriatric medicine and other DGH specialities. When including use of GP beds, practices with access used 6.1% more beds per 1000 patients. Authors conclude that the finding replicate those found in Ref 17339 and 774. Number of possible explanations and proposals for further research given. |
| [104] | Steiner, A., Walsh, B., Pickering, R. M., Wiles, R., Ward, J., Brooking, J. I., and Torgerson, D. J. Therapeutic nursing or unblocking beds? A randomised controlled trial of a post-acute intermediate care unit | 2001 | England | RCT | Patient data | Southampton has an urban teaching hospital and surrounding rural area with 9 community hospitals. The nurse-led intermediate care unit was part of the teaching hospital and located near the main hospital site in a smaller setting 'emphasising outpatient care'. Patients were referred from general medical wards of the hospital and assessed. Must be adult (> 16 years), medically stable for > 24 hours. | To compare intermediate post-acute care in an in-patient nurse-led unit with conventional post-acute care on a general medical ward. | The nurse-led unit had similar outcomes to a conventional medical ward. To make the best use of additional resources, a whole-systems assessment of local responsibilities will be required. |
| [105] | Taubert, G., Bergmeier, C., Andresen, H., Senges, J., and Potratz, J Clinical profile and management of heart failure: rural community hospital vs. metropolitan heart centre | 2001 | Germany | Observational | Cohort | Community hospital set in a sparsely populated rural area in NW Germany providing medical and non-invasive cardiology procedures (pop 80,000). The heart centre is located in a densely populated area of SW Germany (pop 300,000). No details of staffing at the community hospital. | To compare baseline characteristics and management of patients presenting with heart failure due to impaired left ventricular function in a community hospital and a metropolitan heart centre. | The rural hospital performed significantly less of the heart failure management recommended by current guidelines. Strategies to improve heart failure management according to guidelines are urgently required. |
| [106] | Thorne, C. P., Seamark, D. A., Lawrence, C., and Gray, D. J. The influence of general practitioner community hospitals on the place of death of cancer patients | 1994 | England | Cross-sectional survey | patient data | Exeter Health District (population 315,000). Deaths from cancer over one year = 1022 patients of GPs in Health District. 12 GP CHs with total of 428 GP beds. 58% of GPs with access to CHs. | To determine the association between access to GP beds and place of death in cancer patients. | Place of death amongst patients of GPs with access to CHs = Home 29% and CH 41%. No access to CHs = Home 39% and specialist units 39%. Authors conclude that CHs have a role to play in terminal care of cancer patients and access to CHs is associated with a significant reduction in deaths at home or in specialist units |
| [107] | Treasure, R. A. and Davies, J. A. Contribution of a general practitioner hospital: a further study | 1990 | Wales | Audit | Hospital data | Rural GP practice which staffs GP hospital in Brecon, mid-Wales; 20,000 patients living in area. Has 34 general beds, 6 bed maternity unit. Half patient population lives with 1mile of hospital, remainder scattered over 650km2 area. Each GP has speciality: general surgeon, obstetrician & gynaecologist; ear, nose & throat surgeon, one general physician, 2 anaesthetists, paediatrician, and one partner with interest in orthopaedics. | To audit workload of a GP hospital and to compare results with an earlier study (see component paper ref) | Authors conclude GP hospital deals with considerably lager proportion of admissions & outpatient attendances of patients than previous study in 1971; eases burden on local district general hospital at a reasonable cost. (costs are not reported in this study) |
| [108] | Tucker, H. Community hospitals. Progress of a concept of care | 1987 | England | Commentary / Non-systematic review | Literature | Data concerns total of 249 communities hospitals (9,050 beds) in 14 English regions. | Assessing role of community hospitals 15 yrs after their creation | Author concludes community hospital provides breadth of locally available services and focus for professionals and community attracting high degree of practical and financial support. Increasing amount of respite care and rehabilitation services on offer is indicator of role community hospital is currently fulfilling. Highlights some concern over low remuneration for increased workload, training and management responsibilities of GPs |
| [109] | Urquhart, J., Graham, B. J., Ward, A., and Greig, H. Information for community hospitals | 2002 | Scotland |  |  | Applies to Scotland as a whole | 1) Interview staff from community hospitals to determine current use of data and access to data 2) Investigate the quality of data collected 3) Design and test a data collection system 4) Pilot data collection system | Data currently collected did not meet needs of staff, particular problems in collecting diagnostic data. Proposed data collection system piloted in 4 community hospitals. Authors state that the use of READ coding confers a number of advantages in collecting diagnostic data and problems that arose in the feasibility study were mainly associated with the data collection being a paper emulation of a computer based system. |
| [110] | Victor, C. R. End of an experiment: report from an inner city community hospital | 1988 | England | Survey | Questionnaire Hospital data | Inner London City Community Hospital (Paddington & North Kensington Health Authority); 24 beds, admitting 5 main categories of patient: acute medical, observation, convalescence, rehabilitation and carer relief. Excluded: children <16 yrs, obstetric & psychiatric patients, patients with anticipated stay of >28 days. GP responsible for patients & admission & proved 24 hr medical cover; 24 nurses. No outpatient or diagnostic facilities and no consultants. Hospital is within 2 miles of 2 general hospitals, one of which is major teaching hospital | Role & function of urban community hospital (over period of one year) |  |
| [111] | Williams, J. and Last, S. Intermediate care: smoothing the road to recovery | 1998 | England | Descriptive | N/A | Nurse-led intermediate care in 28-bed community hospital in Liverpool. Serves local community and acute trusts. Provides inpatient care for patients of local GPs for clinical respite, rehabilitation and palliative care plus convalescence from acute hospitals. Hospital used to have focus on social care. Run by nurses from wide and varied background (nurse practitioners, associated practitioners, practitioners assistants). Nurse visits after referral, strict eligibility criteria, access to part-time medical officer. | To describe the running of a nurse-led intermediate care unit in a community hospital in Liverpool | The service that has been created provides a high -quality service valued by patients and staff and responsive to local pressures and needs. The service demonstrated innovative thinking and a determination to avoid ritualistic thinking and professional tribalism. |
| [112] | Young, J. and Donaldson, K. Community hospitals and older people | 2001 | UK | Commentary / Non-systematic review | Literature | UK community hospitals in general. | No aims stated. Review of literature. Emphasis on care of older people in community hospitals. Also reviews definition of CH, numbers, roles and functions. | CHs are poorly described in literature. Too few evaluation studies have been conducted. Author concludes direct acute admission for older patients is not appropriate but role of CHs in post acute care could be extended (especially amongst frailer older people or those with fractured neck of femur) |
| [113] | Zaman, S. Geriatricians working in the community. | 2000 | UK | Commentary / Non-systematic review | Literature / expert | Integration of services between geriatricians and community staff including role of CH. | Reviews the potential role of geriatricians in supporting community care. | Calls for a shift in focus from equity of access to acute services for the elderly towards a preventive approach with assessment rather than crisis response. The role of geriatricians in PCT planning and contribution to community care is essential. The role of isolated community geriatrician posts is not supported. |

References

1. Aaraas I, Melbye H, Eriksen BO, Irtun O: **Is the general practitioner hospital a potential 'patient trap'? A panel study of emergency cases transferred to higher level hospitals.** *Scand J Prim Health Care* 1998, **16:** 76-80.

2. Aaraas I, Sorasdekkan H, Kristiansen IS: **Are general practitioner hospitals cost-saving? Evidence from a rural area of Norway.** *Fam Pract* 1997, **14:** 397-402.

3. Anonymous: **Return of cottage hospitals in search for more beds.** *British Journal of Nursing* 2000, **9:** 191.

4. Anthony D, Brooks N: **Clinical guidelines in community hospitals.** *NT Research* 2001, **6:** 839-852.

5. Antrobus M: **Development work in community hospitals.** *Primary Health Care* 1996, **6:** 10-13.

6. Antrobus M: **Professional viewpoint. Community hospital nurses: raising the profile.** *British Journal of Community Health Nursing* 1996, **1:** 307-308.

7. Archibald G: **Patients who have had a stroke: where should their needs be met?** *British Journal of Therapy & Rehabilitation* 1998, **5:** 8-10.

8. Armstrong IJ, Haston WS: **Medical decision support for remote general practitioners using telemedicine.** *Journal of Telemedicine & Telecare* 1997, **3:** 27-34.

9. Ashworth M, Nafisa MA, Corkery M: **Respite care in an intermediate care centre: the views of patients and carers.** *Health & Social Care in the Community* 1996, **4:** 234-245.

10. Baker J, Goldacre M, Muir-Gray JA: **Community hospitals in Oxfordshire: Their effect on the use of specialist inpatient services.** *Journal of Epidemiology & Community Health* 1986, **40:** 117-120.

11. Barker LC, McCarthy ST: **Geriatric day hospitals: consultant and community units compared.[see comment].** *Age & Ageing* 1989, **18:** 364-370.

12. Baxter E, Bushell A, Pearson V: **Community hospitals. Delivering the goods.** *Health Serv J* 1998, **108:** 30.

13. Henderson J. Benefits and costs of community and long-stay health services in the Borders. SOAP 10. 1986. Aberdeen, HERU, University of Aberdeen. HERU Series of Option Appraisal Papers.

14. Berkeley JS: **The role of the cottage hospital in the UK.** *Ecology of Disease* 1983, **2:** 211-214.

15. Brebner EM, Brebner JA, Ruddick-Bracken H, Wootton R, Ferguson J, Palombo A *et al*.: **Evaluation of an accident and emergency teleconsultation service for north-east Scotland.** *Journal of Telemedicine & Telecare* 2004, **10:** 16-20.

16. Brockbank J: **What relevance do community hospital beds have for palliative care patients?** *European Journal of Palliative Care* 2002, **9:** 164-166.

17. Brooks N: **Length of stay in community hospitals.** *Nursing Standard* 2001, **15:** 33-38.

18. Carson M, Close J: **A team approach to the audit of nutritional care in community hospitals.** *Journal of Human Nutrition & Dietetics* 1996, **9:** 309-317.

19. Cavanagh AJ: **Community hospitals.** London: Royal College of General Practitoners; 1995:33-35.

20. Cavenagh AJ, Phillips KM, Sheridan B, Williams EM: **Contribution of isolated general practitioner maternity units.** *British Medical Journal Clinical Research Ed* 1984, **288:** 1438-1440.

21. Church J, Seamark D: **A survey of surgical activity in UK community hospitals.** *Annals of the Royal College of Surgeons of England* 2002, **84:** 111-112.

22. Coast J, Inglis A, Frankel S: **Alternatives to hospital care: what are they and who should decide?** *Br Med J* 1996, **312:** 162-166.

23. Coast J, Inglis A, Morgan K, Gray S, Kammerling M, Frankel S: **The Hospital admissions study:are there alternatives to emergency hospital admissions?** *Journal of Epidemiology and Community Health* 1995, **49:** 194-199.

24. Cook PJ, Porter L: **Community hospitals and district general hospital medical bed use by elderly people: A study of 342 general practitioner beds in Oxfordshire.** *Age & Ageing* 1998, **27:** 357-361.

25. Cormie P. Palliative care in community hospitals in the Borders. Scottish Borders Palliative and Cancer Care Site . 1999.

26. Dick RW: **Inquest on QUEST.** *International Journal of Health Care Quality Assurance* 1996, **9:** 10-15.

27. Donald IP, Jay T, Linsell J, Foy C: **Defining the appropriate use of community hospital beds.** *British Journal of General Practice* 2001, **51:** 95-100.

28. Ebmeier KP, Hunter D, Beattie JAG, Eagles JM, Blackwood GW, Besson JAO *et al*.: **Continuing care of the demented elderly in Inverurie.** *Health Bull* 1988, **46:** 32-41.

29. Edwards N: **Models of perfection... small general hospitals will have to adapt and change.** *Health Serv J* 1998, **108:** 24-26.

30. Garrett T, House W, Lowe SW: **Outcome of women booked into an isolated general practice maternity unit over eight years.** *Journal of the Royal College of General Practitioners* 1987, **37:** 488-490.

31. George J: **Community hospitals...and district general hospitals [7].** *Age & Ageing* 1999, **28:** 240.

32. Grant J, Ramsay A, Bain J: **Community hospitals and general practice: extended attachments for medical students.** *Medical Education* 1997, **31:** 364-368.

33. Grant JA, Dowell J: **A qualitative study of why general practitioners admit to community hospitals.** *British Journal of General Practice* 2002, **52:** 628-630.

34. Grant JA: **Community hospitals--time to come off the fence.[see comment].** *Journal of the Royal College of General Practitioners* 1989, **39:** 226-227.

35. Grant JA: **Contribution of general practitioner hospitals in Scotland.** *Br Med J* 1984, **288:** 1366-1368.

36. Griffiths PD, Edwards ME, Forbes A, Harris RL, Ritchie G: **Effectiveness of intermediate care in nursing-led in-patient units (protocol).** *The Cochrane Library* 2004.

37. Griffiths RA, Wilcock GK: **Geriatric medicine in two community hospitals - The Oxford experience.** *Journal of Clinical & Experimental Gerontology* 1981, **3:** 399-409.

38. Hall R, Ramaiah RS, Jones DT: **General practitioner hospitals: coming or going?** *Br Med J* 1984, **288:** 1691.

39. Halpin SF, Yeoman L, Dundas DD: **Radiographic examination of the lumbar spine in a community hospital: an audit of current practice.[see comment].** *Br Med J* 1991, **303:** 813-815.

40. Hamilton W, Round A: **Urban community hospitals.** *British Journal of General Practice* 1995, **45:** 326-327.

41. Harris G: **The context and content of community hospital admissions.** *Journal of the Royal College of General Practitioners* 1986, **36:** 363-365.

42. Hawkins A: **Triage in rural hospitals.** *Australian Emergency Nursing Journal* 2000, **3:** 19-20.

43. Haynes RM, Bentham CG: *Community hospitals and rural accessibility*, 1981 edn. Farnborough: Saxon House; 1981.

44. Haynes R, Gale S, Mugford M, Davies P: **Cataract surgery in a community hospital outreach clinic: patients' costs and satisfaction.** *Soc Sci Med* 2001, **53:** 1631-1640.

45. Heard-Dimyan J: **Issue of emergency hormonal contraception through a casualty department in a community hospital.** *British Journal of Family Planning* 1999, **25:** 105-109.

46. Hegney D, McCarthy A, Pearson A: **Effects of size of health service on scope of rural nursing practice.** *Collegian* 1999, **6:** 21-26.

47. Henderson LR, Scott A: **The costs of caring for stroke patients in a GP-led community hospital: an application of programme budgeting and marginal analysis.** *Health & Social Care in the Community* 2001, **9:** 244-254.

48. Higgins J: **Community hospitals. Cottage industry.** *Health Serv J* 1994, **104:** 30-31.

49. Higgs R: **Example of intermediate care: the new Lambeth Community Care Centre.** *Br Med J* 1985, **291:** 1395-1397.

50. Hindle H, Norheim JK, Renger R: **Rural Alberta thrombolysis study. Survey of practice patterns for managing acute myocardial infarction.** *Canadian Family Physician* 1995, **41:** 1180-1187.

51. Hine C, Wood VA, Taylor S, Charny M: **Do community hospitals reduce the use of district general hospital inpatient beds?** *Journal of the Royal Society of Medicine* 1996, **89:** 681-687.

52. ISD. Community hospitals information project: final report. 2001. Edinburgh, ISD.

53. James AM: **Closing rural hospitals in Saskatchewan: on the road to wellness?** *Social Science and Medicine* 1999, **49:** 1021-1034.

54. Jarman B: **Community hospitals: a desirable option?** *Quality in Health Care* 1995, **4:** 160.

55. Jeavons R, Taylor P. Review of community hospitals: a report prepared for Scarborough Health Authority. 1989. York, University of York Health Economics Consortium.

56. Jeffrey D: **Education in palliative care: a qualitative evaluation of the present state and the needs of general practitioners and community nurses.** *European Journal of Cancer Care* 1994, **3:** 67-74.

57. Johnson DB: **Audit of surgical practice in a community hospital.** *British Medical Journal Clinical Research Ed* 1984, **288:** 1293-1295.

58. Jones R, Tucker H: **The role of community hospitals.** *Health Trends* 1988, **20:** 45-48.

59. Jones R: **General practitioner beds in Finland - lessons for the UK?** *Journal of the Royal College of General Practitioners* 1986, **37:** 28-30.

60. Jones R: **Do general practitioner hospitals extend primary care?** *British Medical Journal Clinical Research Ed* 1986, **292:** 243-244.

61. Kerrin D, Jones R: **Community hospitals.[comment].** *Journal of the Royal College of General Practitioners* 1989, **39:** 434.

62. Latimer J: **Nursing in a different way.** *Sr Nurse* 1987, **6:** 28-29.

63. Legge A: **Nurse-led hospital service takes on GPs' night calls.** *Nurs Times* 1998, **94:** 57.

64. Lemaire ED, Boudrias Y, Greene G: **Low-bandwidth, Internet-based videoconferencing for physical rehabilitation consultations.** *Journal of Telemedicine & Telecare* 2001, **7:** 82-89.

65. Licence K, Crichton C, Stark C: **Changes in the pattern of work in a rural community hospital 1987-1997.** *Health Bull* 1999, **57:** 312-317.

66. Liddell R, Grant J, Rawles J: **The management of suspected myocardial infarction by Scottish general practitioners with access to community hospital beds.[see comment].** *British Journal of General Practice* 1990, **40:** 318-322.

67. Light D, Dixon M: **Intermediate care: a new way through.** *Health Serv J* 2000, **10:** 24-25.

68. Liu L, Hader J, Brossart B, White R, Lewis S: **Impact of rural hospital closures in Saskatchewan, Canada.** *Social Science and Medicine* 2001, **52:** 1793-1804.

69. Llewellyn J, Evans N, Walsh H: **Service provision. The role of the community hospital in the care of dying people.** *International Journal of Palliative Nursing* 1999, **5:** 244-249.

70. Lloyd-Williams M: **Survey of palliative care in a general practitioner unit.** *Journal of Cancer Care* 1996, **5:** 97-99.

71. Lowe SW, House W, Garrett T: **Comparison of outcome of low-risk labour in an isolated general practice maternity unit and a specialist maternity hospital.** *Journal of the Royal College of General Practitioners* 1987, **37:** 484-487.

72. Lyon A, Love DR: **Terminal care: the role of the general practitioner hospital.** *Journal of the Royal College of General Practitioners* 1984, **34:** 331-333.

73. Mabrook AF, Dale B: **Can nurse practitioners offer a quality service? An evaluation of a year's work of a nurse led minor injury unit.[see comment].** *Journal of Accident & Emergency Medicine* 1998, **15:** 266-268.

74. Macduff C, West BJM, Lawton S: **An evaluation of the impact of developing nurse-led treatments for minor injuries in community hospital casualty units... including commentary by Dolan B.** *NT Research* 2000, **5:** 276-285.

75. McConnon JK: **The Canadian community hospital.** *Lancet* 1987, **1:** 266-267.

76. McCormack B: **Community hospital-issues for older people.** *Elderly Care* 1998, **10:** 42-43.

77. McCormack B: **The developing role of community hospitals: an essential part of a quality service.** *Quality in Health Care* 1993, **2:** 253-258.

78. McKinlay WJD: **Why we need community hospitals for the elderly.** *Geriatric Medicine* 1991, **21:** 11-12.

79. Meads G: **Rediscovering community hospitals.** *British Journal of General Practice* 2001, **51:** 91-92.

80. North NT, Hall DJ, Kearns WE: **First year of an inner city general practitioner community hospital.** *British Medical Journal* 1984, **288:** 1209-1211.

81. Palombo A, Ferguson J, Rowlands A, Pedley D, Fraser S: **An evaluation of a telemedicine fracture review clinic.** *Journal of Telemedicine & Telecare* 2003, **9:** 31-33.

82. Payne S, Ramaiah RS: **The importance of general practitioner day hospitals.** *Practitioner* 1986, **230:** 199-201.

83. Pedley D, Ferguson J, Palombo A, Richardson J: **Community coronary units: strategies to promote pre-hospital thrombolysis.** *Journal of Telemedicine & Telecare* 2002, **8:** 24-25.

84. Pencheon D: **Intermediate care.** *Br Med J* 2002, **324:** 1347-1348.

85. Primrose WR: **Community hospitals.** *Age & Ageing* 1998, **27:** 261-263.

86. Ramaiah S: **Community hospitals in the new NHS.[see comment].** *BMJ* 1994, **308:** 487-488.

87. Ritchie LD, Robinson K: **Community hospitals: new wine in old bottles?** *British Journal of General Practice* 1998, **48:** 1039-1040.

88. Round A, Crabb T, Buckingham K, Mejzner R, Pearce V, Ayres R *et al*.: **Six month outcomes after emergency admission of elderly patients to a community or a district general hospital.** *Fam Pract* 2004, **21:** 173-179.

89. Royal College of General Practitioners: *Community hospitals: preparing for the future.* London: Royal College of General Practitioners; 1990.

90. Salmon S, Brint G, Marshall D, Bradley A: **Telemedicine use in two nurse-led minor injuries units.** *Journal of Telemedicine & Telecare* 2000, **6:** S43-S45.

91. Sanger R, Clyne CA: **The surgical value of community hospitals: a closer look.** *Annals of the Royal College of Surgeons of England* 1991, **73:** 77-80.

92. Scottish Partnership for Palliative Care. Palliative care in community hospitals: report of a working party of the Scottish Partnership Agency for Palliative and Cancer Care. 1998. Scottish Partnership for Palliative Care.

93. Seamark DA, Williams S, Hall M, Lawrence CJ, Gilbert J: **Dying from cancer in community hospitals or a hospice: closest lay carers' perceptions.** *British Journal of General Practice* 1998, **48:** 1317-1321.

94. Seamark DA, Williams S, Hall M, Lawrence CJ, Gilbert J: **Palliative terminal cancer care in community hospitals and a hospice: a comparative study.** *British Journal of General Practice* 1998, **48:** 1312-1316.

95. Seamark D, Moore B, Tucker H, Church J, Seamark C: **Community hospitals for the new millennium.** *British Journal of General Practice* 2001, **51:** 125-127.

96. Seamark D, Seamark C, Lawrence C: **Contribution of community hospitals in East Devon to cancer workload.** *Health Trends* 1997, **29:** 114-117.

97. Shacklady J, Browne M: **Community hospitals. Cornish hops.** *Health Serv J* 1999, **109:** 26-27.

98. Shaw CD, Collins CD: **Health service accreditation: report of a pilot programme for community hospitals.** *Br Med J* 1995, **310:** 781-784.

99. Shaw CD: **General practitioner hospitals: coming or going?** *Br Med J* 1984, **288:** 1399.

100. Anonymous: **Simple solutions: clinical training for the whole staff: rural hospital puts all staff at the bedside.** *Healthcare Benchmarks* 2002, **9:** 56-57.

101. Smith K: **Distance learning.** *Health Serv J* 2002, 14-15.

102. Smith M: **The development of rehabilitation for older people in a community hospital.** *Therapy weekly* 2004, **30:** 11-14.

103. Stark C, Oliver K, Hopkins P: **Effect of general practitioner hospitals on district general hospital bed use in the Highlands of Scotland.** *Health Bull* 2000, **58:** 385-389.

104. Steiner A, Walsh B, Pickering RM, Wiles R, Ward J, Brooking JI *et al*.: **Therapeutic nursing or unblocking beds? A randomised controlled trial of a post-acute intermediate care unit.** *Br Med J* 2001, **322:** 453-460.

105. Taubert G, Bergmeier C, Andresen H, Senges J, Potratz J: **Clinical profile and management of heart failure: rural community hospital vs. metropolitan heart center.** *European Journal of Heart Failure* 2001, **3:** 611-617.

106. Thorne CP, Seamark DA, Lawrence C, Gray DJ: **The influence of general practitioner community hospitals on the place of death of cancer patients.** *Palliat Med* 1994, **8:** 122-128.

107. Treasure RA, Davies JA: **Contribution of a general practitioner hospital: a further study.** *Br Med J* 1990, **300:** 644-646.

108. Tucker H: **Community hospitals. Progress of a concept of care.** *Health Service Journal* 1987, **97:** 244-245.

109. Urquhart J, Graham BJ, Ward A, Greig H: **Information for community hospitals.** *Health Bulletin* 2002, **60:** 13-19.

110. Victor CR: **End of an experiment: report from an inner city community hospital.** *Journal of the Royal College of General Practitioners* 1988, **38:** 407-410.

111. Williams J, Last S: **Intermediate care: smoothing the road to recovery.** *Nurs Times* 1998, **94:** 52-54.

112. Young J, Donaldson K: **Community hospitals and older people.** *Age & Ageing* 2001, **30:** 7-10.

113. Zaman S: **Geriatricians working in the community.** *CME Bulletin Geriatric Medicine* 2000, **2:** 44-47.
